# Supplementary material for: ACTR5 controls CDKN2A and tumor progression in an INO80-independent manner
Source: Sci Adv. 2022 Dec 23;8(51):eadc8911. doi: 10.1126/sciadv.adc8911 (PMC9788768; doi:10.1126/sciadv.adc8911)
Supplement: 20221223-1 [file sciadv.adc8911.v1.pdf]

CANCER

ACTR5 controls CDKN2A and tumor progression in an INO80-independent manner

Copyright © 2022 The Authors, some rights reserved; exclusive licensee American Association for the Advancement of Science. No claim to original U.S. Government Works. Distributed under a Creative Commons Attribution License 4.0 (CC BY).

Xiaobao Xu[1], Anthony K. N. Chan[1], Mingli Li[1], Qiao Liu[1], Nicole Mattson[1], Sheela Pangeni Pokharel[1], Wen-Han Chang[1], Yate-Ching Yuan[2], Jinhui Wang[2], Roger E. Moore[2], Patrick Pirrotte[2,3], Jun Wu[2], Rui Su[1,2], Markus Müschen[4], Steven T. Rosen[2], Jianjun Chen[1,2], Lu Yang[1], Chun-Wei Chen[1,2]*

Epigenetic dysregulation of cell cycle is a hallmark of tumorigenesis in multiple cancers, including hepatocellular carcinoma (HCC). Nonetheless, the epigenetic mechanisms underlying the aberrant cell cycle signaling and therapeutic response remain unclear. Here, we used an epigenetics-focused CRISPR interference screen and identified ACTR5 (actin-related protein 5), a component of the INO80 chromatin remodeling complex, to be essential for HCC tumor progression. Suppression of ACTR5 activated CDKN2A expression, ablated CDK/E2F-driven cell cycle signaling, and attenuated HCC tumor growth. Furthermore, high-density CRISPR gene tiling scans revealed a distinct HCC-specific usage of ACTR5 and its interacting partner IES6 compared to the other INO80 complex members, suggesting an INO80-independent mechanism of ACTR5/IES6 in supporting the HCC proliferation. Last, our study revealed the synergism between ACTR5/IES6-targeting and pharmacological inhibition of CDK in treating HCC. These results indicate that the dynamic interplay between epigenetic regulators, tumor suppressors, and cell cycle machinery could provide novel opportunities for combinational HCC therapy.

INTRODUCTION

Liver cancer is one of the most frequently diagnosed cancer and the overall third leading cancer-related death with a stunning 0.83 million people dying from liver cancer in 2020 (*1*). Among primary liver cancer, hepatocellular carcinoma (HCC) accounts for approximately 90% of all cases with a 5-year survival of 18% (*2*). While the early-stage HCC tumor might be curable via surgical interventions (liver resection, transplantation, and ablation) (*3*), patients with advanced HCC who depend on systemic treatments exhibit a poor prognosis of median survival of 6.5 to 10.7 months despite the use of targeted therapies (e.g., the multi-kinase inhibitor sorafenib) (*4*). The prevalence of these hepatic disorders and the lack of effective treatments highlight the critical need for more effective therapeutic strategies.

Epigenetics represent heritable mechanisms of controlling gene functions without changes in the underlying DNA sequence, which are orchestrated by repertoires of nuclear proteins modulating DNA/histone modifications, nucleosome positioning, chromatin remodeling, etc. (*5*). Epigenetic dysregulation has been implicated in chronic liver disorders and HCC tumorigenesis (*6*, *7*). Since liver is a highly regenerative organ, the robust proliferative capacity required for tissue homeostasis is often skewed by HCC to support aggressive cancer growth (*8*–*10*). Epigenetic lesions at cell cycle regulators and tumor suppressor genes such as the silencing of *cyclin-dependent kinase inhibitor 2A* (*CDKN2A*) locus are frequently found in patients with HCC (*11*–*14*). The impaired cell cycle checkpoints in HCC could trigger cascades of molecular alternations, including the activation of CDKs (e.g., CDK4/6) and E2F transcription factors (e.g., E2F1), driving an uncontrollable proliferation of the HCC cells (*15*, *16*). Therefore, the identification of critical epigenetic mechanisms required for the expansion/maintenance of HCC represents an attractive research field for future therapeutic development against liver malignancies.

In this study, we performed a custom CRISPR interference (CRISPRi) (*17*) library screen in HCC cells that targets more than 700 epigenetic regulator genes in the human genome. Distinct from the commonly used genome-wide CRISPR knockout (KO) screens, our epigenetic-focused CRISPRi screen acted through inhibition of target gene expression. As a result, our screen identified a previously unknown effector actin-related protein 5 (ACTR5; also known as ARP5) (*18*) in HCC, which was not revealed in the large-scale CRISPR-KO screen consortium databases (*19*, *20*). Using transcriptomics [RNA sequencing (RNA-seq)], epigenetics [chromatin immunoprecipitation sequencing (ChIP-seq)], and proteomics [liquid chromatography tandem mass spectrometry (LC-MS/MS)] profiling followed by validation assays, our study collectively revealed that ACTR5 is required for CDKN2A silencing and CDK6/E2F1-mediated cell cycle progression in HCC. We also used a high-density CRISPR tiling screen approach (*21*–*23*) and identified the critical domains in ACTR5 for interacting with its partner IES6 (also known as INO80 complex subunit C), which is crucial for stabilizing ACTR5 protein and maintaining HCC proliferation. While the ACTR5/IES6 has been frequently described as a module of the INO80 chromatin remodeling complex (*24*, *25*), our results revealed a distinct role of ACTR5/IES6 from the main INO80 subunits (e.g., INO80, MCRS1, ACTR8, and YY1). The selective dependency in HCC emphasizes an INO80-independent role of ACTR5/IES6 in HCC maintenance, cell cycle control, and therapeutic response.

[1]Department of Systems Biology, Beckman Research Institute - City of Hope, Duarte, CA, USA. [2]City of Hope Comprehensive Cancer Center, Duarte, CA, USA. [3]Cancer and Cell Biology Division, Translational Genomics Research Institute (TGen), Phoenix, AZ, USA. [4]Center of Molecular and Cellular Oncology, Yale Cancer Center, Yale School of Medicine, New Haven, CT, USA.
*Corresponding author. Email: cweichen@coh.org

RESULTS

CRISPRi screen identifies ACTR5 as a novel vulnerability in HCC

To characterize critical epigenetic mechanisms supporting the maintenance of HCC, we used a custom CRISPRi library containing a total of 3669 single guide RNAs for CRISPRi (sgiRNAs) targeting the transcription start site (TSS) of 728 epigenetic-related genes in the human genome (Fig. 1A, fig. S1, and data S1). We delivered this library into the HepG2 cells stably expressing an enzymatic-inactivated Cas9 fusion with the transcription repressor Krab (i.e., HepG2-dCas9-Krab cells; fig. S2) using lentiviral transduction and compared the frequency change of each integrated single guide RNA (sgRNA) construct in these cells between days 0 and 24 using high-throughput sequencing followed by the Model-based Analysis of Genome-wide CRISPR-Cas9 KO (MAGeCK) algorithm (Fig. 1B and data S2) (*26*). In addition to the positive controls (sgRNA targeting genes commonly essential to cancer cells; red dots), we observed six candidate essential genes in HCC from the screen (blue dots). Evaluation of the gene dependency score (z score) over 22 HCC cell line models in the genome-wide CRISPR screen database (DepMap; The Cancer Dependency Map Project, Broad Institute; data S3) (*19*, *20*) revealed that five of these candidate genes (*WDR5*, *SMC1A*, *DDX23*, *SF3A1*, and *EFTUD2*) are crucial in HCC (Fig. 1C; median z score ≤ -1). One of the candidates hit from our screen, *ACTR5* (green), was not recognized as an essential gene in HCC in the DepMap database.

To validate our CRISPRi library screen results, we transduced the HepG2 cells with CRISPRi sgRNAs targeting *ACTR5* (sgiACTR5). Using a red fluorescent protein (RFP; co-expressed with sgRNA) flow cytometric growth competition assay (fig. S3A), we found that cells expressing sgiACTR5 were selectively outcompeted compared to cells transduced with nontargeting control sgRNAs (sgiNTC) (Fig. 1D). To examine the impact of ACTR5 depletion over diverse cell types, we CRISPR-targeted *ACTR5* in two additional HCC cell lines SNU182 and SNU475 and compared it to the non-HCC cancer cell types, including acute myeloid leukemia (MV4-11 and MOLM13), breast cancer (MDA-231 and MCF7), and glioblastoma (U251 and U87) (Fig. 1E). Efficient CRISPR editing and cell

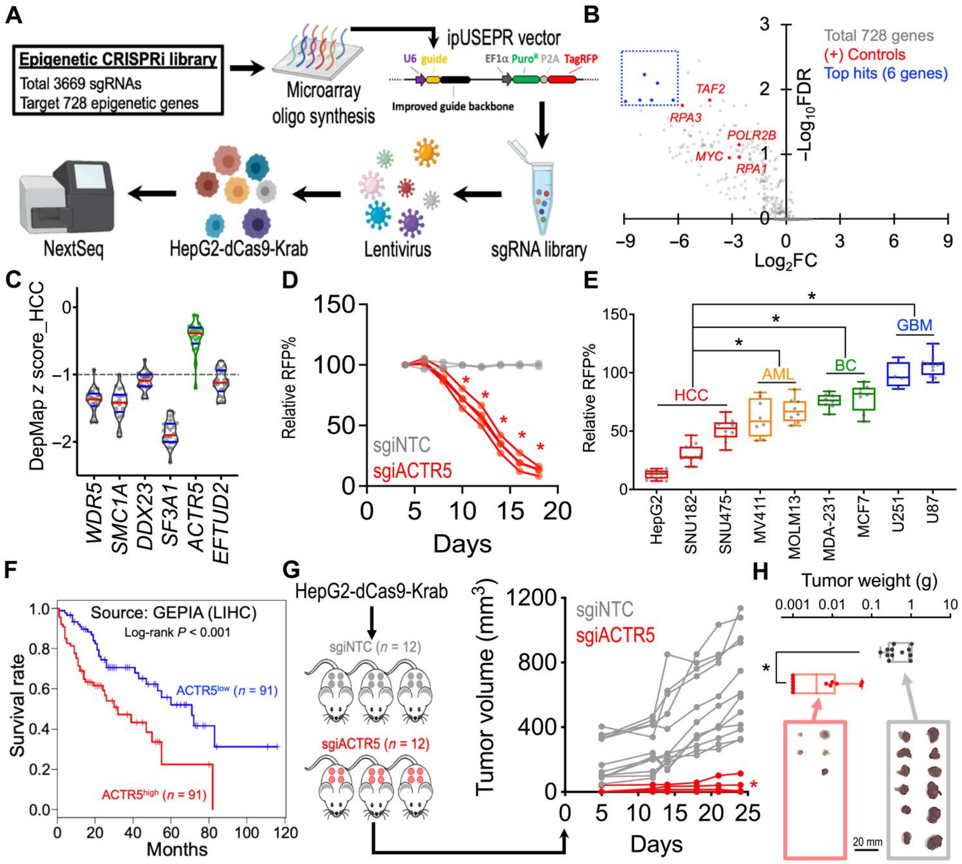

Fig. 1. CRISPRi screen identifies the essential role of ACTR5 in HCC. (**A**) Schematic outline of an epigenetic-focused CRISPRi screen in HepG2-dCas9-Krab cells. (**B**) Volcano plot depicts the change of sgRNA abundance [x axis; $\log_2$ (fold change)] and significance [y axis; $-\log_{10}$ (false discovery rate)] of each gene during the 24-day epigenetics CRISPRi screen. (**C**) Violin plot indicates the median (red lines) and quartiles (blue lines) of the gene dependency score (z score) over 22 HCC cell lines (dots) in the DepMap genome-wide CRISPR screen 20Q2 database. (**D**) Growth competition assay of HepG2-dCas9-Krab cells transduced with RFP-labeled nontargeting control (gray lines; $n = 2$ independent sgiNTC sequences) and ACTR5-targeting sgiRNAs (red lines; $n = 4$ independent sgiACTR5 sequences). (**E**) Box-whiskers plot of the growth competition assay in nine Cas9-expressing cancer cell models transduced with sgACTR5 (dots; $n = 4$ independent sgACTR5 sequences). (**F**) Survival curves of liver HCC patients with high versus low *ACTR5* expression. Source: GEPIA database (http://gepia.cancer-pku.cn). (**G**) Profile plot of tumor volume (in cubic millimeters) and (**H**) box-whiskers plot of tumor weight (in grams) of the HCC xenograft tumors in NSG mice inoculated with sgiNTC and sgiACTR5-transduced HepG2-dCas9-Krab cells ($n = 12$ tumor sites per group). Box-whiskers indicate the first and third quartiles (boxes) and the range (whiskers). *$P < 0.01$ by two-sided Student's t test.

suppression were observed in these nine Cas9-expressing cell models, evidenced by robust depletion of the RFP-positive cells with sgRNA targeting the general cancer essential gene proliferating cell nuclear antigen (PCNA) (fig. S3B). In contrast, depletion of the RFP-positive cells in CRISPR-KO sgRNAs targeting *ACTR5* (sgACTR5)-transduced cultures was significantly more pronounced in HCC than in other cancer cell types (Fig. 1E). Clinically, we observed an association of high *ACTR5* expression level with poor survival prognosis in patients with liver HCC (Fig. 1F; source: Gene Expression Profiling Interactive Analysis) (*27*). Last, suppression of ACTR5 significantly retarded the in vivo HCC tumor progression (Fig. 1G) and reduced the tumor mass (Fig. 1H; sgiNTC = 0.537 ± 0.097 g; sgiACTR5 = 0.013 ± 0.006 g; data represent day 24 mean tumor weight ± SEM) in the HepG2 xenograft model, indicating the indispensable role of ACTR5 in HCC maintenance.

ACTR5 maintains the E2F cell cycle program via inhibiting CDKN2A expression

To elucidate the transcriptomic impact induced by knockdown of ACTR5, we performed RNA-seq and Gene Set Enrichment Analysis (GSEA) (*28*) in HepG2-dCas9-Krab cells transduced with sgiNTC versus sgiACTR5. We found that in HepG2 cells, the "E2F_Pathway" is among the most depleted GSEA hallmark gene sets upon ACTR5 knockdown (Fig. 2A and data S4). To identify genes directly regulated by ACTR5 in HCC, we captured genomic DNA associated with the Twin-Strep–tagged ACTR5 (ACTR5-TST) from HepG2 using the Strep-Tactin XT beads (*29*) for high-throughput sequencing (Fig. 2B) and then overlapped the ACTR5-bound targets (525 genes) with the E2F_Pathway genes (145 genes). Of the 13 overlapped candidates, we identified a substantial induction of CDKN2A mRNA (Fig. 2C and data S5) and protein (Fig. 2D) levels in the sgiACTR5-transduced HepG2 cells. Furthermore, the presence of ACTR5 at the *CDKN2A* promoter region was confirmed by TST-mediated ChIP-seq and ChIP–quantitative polymerase chain reaction (qPCR) (Fig. 2E). While the general histone modification landscapes at the ACTR5-bound genes remain comparable to those at the ACTR5 unbound genes (fig. S4) (*30*), we focused on the levels of two histone modifications, H3K9me2 and H3K27me3, which have been implicated in epigenetic repression of *CDKN2A* (*31*, *32*). Our results revealed a significant reduction of H3K9me2 but not H3K27me3 at the *CDKN2A* TSS locus upon ACTR5 depletion (Fig. 2, F and G), indicating a role of ACTR5 in recruiting H3K9 epigenetic silencing of *CDKN2A* in HCC. Notably, the induction of CDKN2A expression by ACTR5 knockdown was not observed in the CDKN2A-null U87

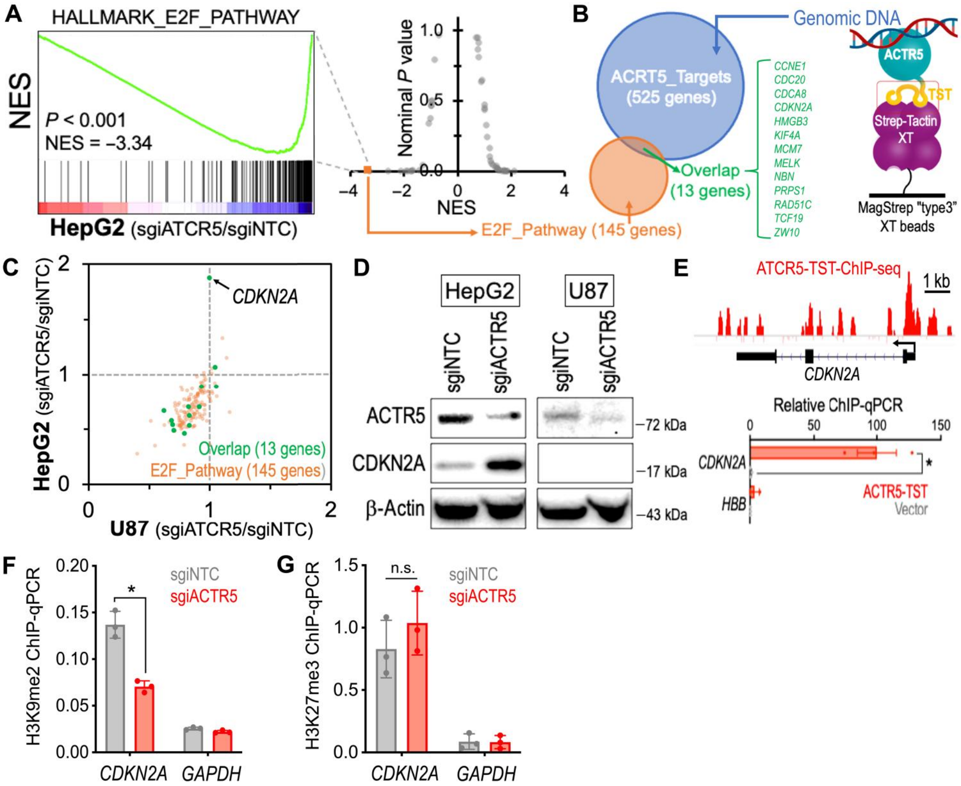

Fig. 2. ACTR5 mediates epigenetic silencing of CDKN2A in HCC. (**A**) RNA-seq and GSEA analyses showing changes in expression of the E2F_Pathway gene set in sgiNTC- versus sgiACTR5-transduced HepG2-dCas9-Krab cells. (Right) Each dot indicates one gene set from the GSEA HALLMARK Database. NES, normalized enrichment score. (**B**) Venn diagram revealed 13 ACTR5-bound target genes within the E2F_Pathway gene set (green). (**C**) RNA-seq expression change of the ACTR5-regulated E2F-Pathway genes (green dots) induced by sgiACTR5 in dCas9-Krab–expressing HepG2 (*y* axis) versus U87 (*x* axis) cells. (**D**) Western blot of ACTR5, CDKN2A, and β-actin in dCas9-Krab–expressing HepG2 and U87 cells transduced with sgiNTC and sgiACTR5. (**E**) TST-mediated ChIP-seq and ChIP-qPCR of ACTR5 at the *CDKN2A* locus in HepG2 cells. (**F**) Level of H3K9me2 and (**G**) H3K27me3 at the *CDKN2A* and *glyceraldehyde-3-phosphate dehydrogenase* (*GAPDH*) loci detected by ChIP-qPCR. Data are presented as means ± SEM. *$P < 0.01$ by two-sided Student's *t* test. n.s., not significant.

cells (Fig. 2, C and D), suggesting a requirement of CDKN2A expression in the ACTR5 dependency.

CDKN2A is a cell cycle suppressor that controls the function of its downstream effectors, including CDK6, Rb (retinoblastoma 1), and E2F1 (*33*). With the induction of CDKN2A expression by sgiACTR5, immunoblotting revealed a drastic reduction of CDK6, phospho-S780 Rb (p-Rb), and E2F1 protein level in the ACTR5-dependent HCC cells (Fig. 3A; HepG2 and SNU475). Consistently, we observed a significant reduction of cells in the S phase in the sgiACTR5-transduced HepG2 cell line (Fig. 3B). Gene ontology analyses using g:Profiler (*34*) also exhibited significant depletions of cell cycle–related genes (fig. S5) and E2F motif–containing genes (fig. S6) in the sgiACTR5-transduced HepG2 cells. In contrast, the expression of CDKN2A and CDK6 was not detected in the ACTR5-independent U251 and U87 cells (Fig. 3A; these glioblastoma cells harbor homozygous deletion at the *CDKN2A* locus) (*35*, *36*). Consequently, the reduced p-Rb and E2F1 levels were not observed in these cell lines. Furthermore, ectopic expression of ACTR5-TST reversed the altered CDKN2A pathway (Fig. 3C) and rescued the proliferation (Fig. 3D) of sgiACTR5-transduced HepG2 cells. Together, our results nominated CDKN2A as the cell cycle checkpoint underlying the ACTR5 network to control tumor progression (Fig. 3E).

CRISPR gene tiling scans revealed an INO80-independent role of ACTR5 in HCC

To investigate whether ACTR5 contains gene regions selectively essential to HCC, we used the high-density CRISPR gene body scan that enables identification of functional elements within a protein by saturation mutagenesis achieved through CRISPR-mediated genome editing (Fig. 4A) (*22*, *23*). First, we developed a pooled library composed of 284 sgRNAs that target every "NGG" protospacer adjacent motifs within the ACTR5-coding exons [targeting density 6.4 base pairs (bp) per sgRNA or 2.1 amino acids per sgRNA]. We then screened this ACTR5 scan library in the Cas9-expressing HepG2 and U87 cells and mapped the results to the ACTR5 peptide position (data S6). Using local smoothened modeling (*37*), this high-resolution genetic screen approach revealed the dependency of HepG2 on multiple regions within the N- and C-terminal actin-fold domains (Fig. 4B, red). The U87 cells were not sensitive to the entire ACTR5 scanning CRISPR library (blue), restating the cell type–specific role of ACTR5 in HCC. Since ACTR5 has been frequently described as a member of the INO80 chromatin remodeling complex (*24*, *25*), we extended the high-density CRISPR gene body scan to examine other vital components of the INO80 complex, including INO80, MCRS1, ACTR8, and YY1 (Fig. 4, C to F, and data S7 to S10). Unexpectedly, none of these INO80 members exhibited a HepG2-selective essential domain, suggesting a distinct usage of ACTR5 in HCC that is unconventional to the INO80 complex.

Comparing the ACTR5 gene body scans in HepG2 and U87 revealed six CRISPR-hypersensitive elements [normalized CRISPR score (NCS) ≤ −1.0] in HepG2 cells (designated A1 to A6; Fig. 4B and fig. S7). Modeling of the ACTR5 three-dimensional structure by AlphaFold (*38*) revealed that five of the six CRISPR scan hit regions were involved in the structural backbone of ACTR5 [Fig. 4G; including A1 (D114-D129), A2 (P159-Y172), A3 (L246-H261), A4 (I449-Q465), and A6 (I552-C569)]. On the other hand, one CRISPR-hypersensitive region, A5 (G502-S519; located within the C-terminal actin-fold domain), was predicted

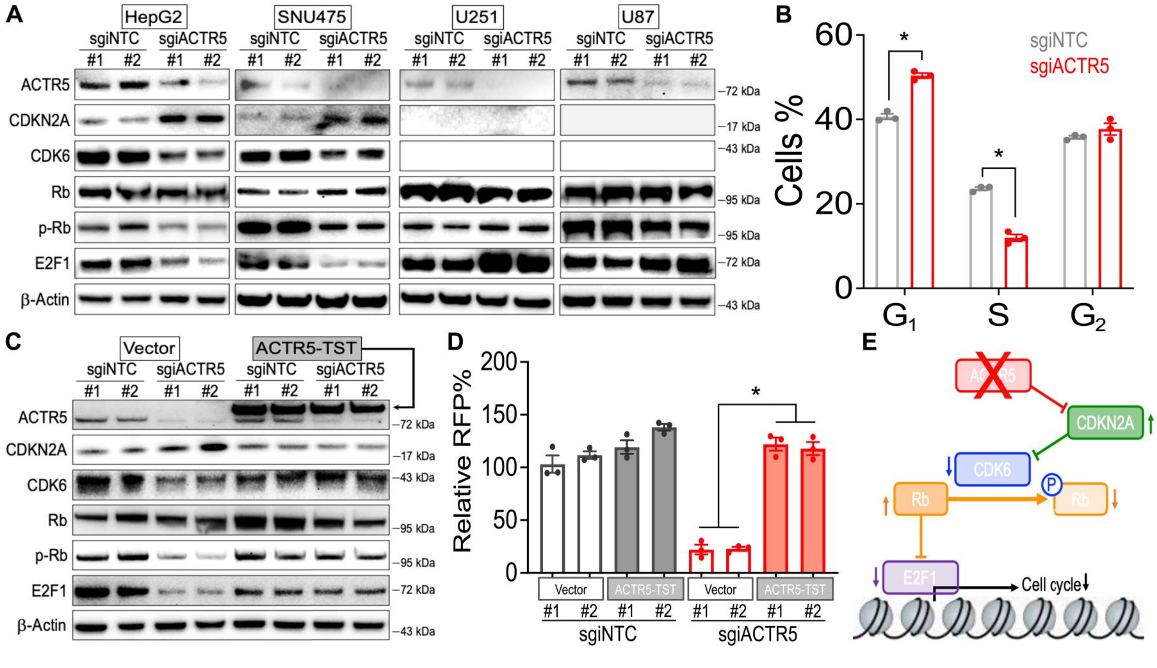

Fig. 3. ACTR5 controls the CDKN2A and CDK6 cell cycle signaling in HCC. (**A** and **C**) Western blot of ACTR5, CDKN2A, CDK6, Rb, p-Rb, E2F1, and β-actin in (A) dCas9-Krab–expressing HCC (HepG2 and SNU475) and glioblastoma (U251 and U87) cells, and (C) vector versus ACTR5-TST–expressing HepG2-dCas9-Krab cells transduced with sgiNTC and sgiACTR5. (**B**) Cell cycle monitored by 5-Ethynyl-2′-deoxyuridine incorporation in HepG2-dCas9-Krab cells transduced with sgiNTC and sgiACTR5 (*n* = 3). (**D**) Growth competition assay of vector versus ACTR5-TST–expressing HepG2-dCas9-Krab cells transduced with RFP-labeled sgiNTC and sgiACTR5 (*n* = 3 each group). (**E**) Effect of targeting ACTR5 on CDKN2A- and CDK6-triggered cell cycle signaling. Data are presented as means ± SEM. *$P < 0.01$ by two-sided Student's *t* test.

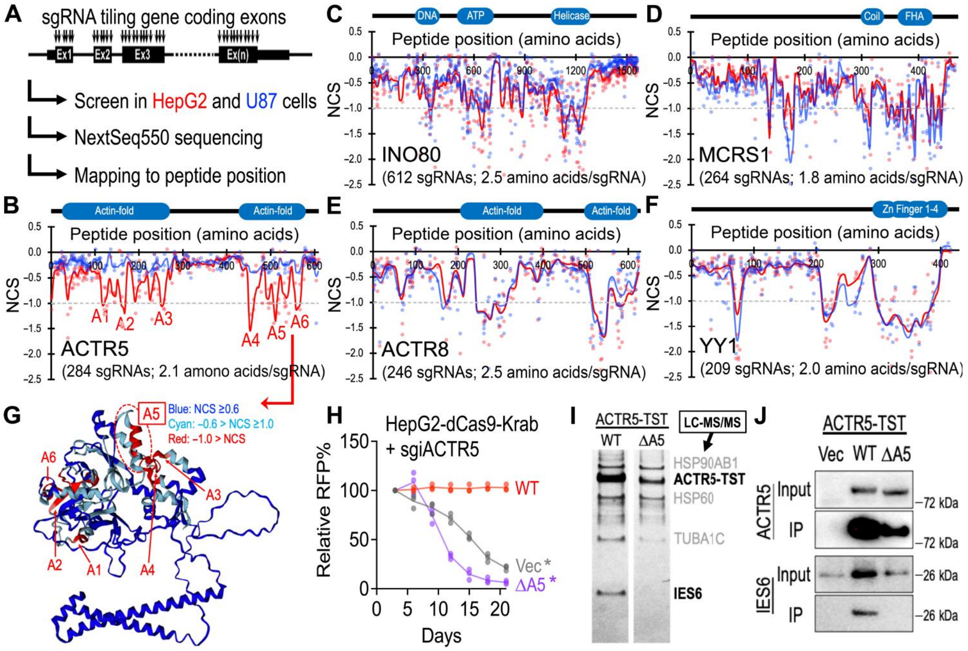

Fig. 4. CRISPR gene tiling scans of INO80 complex members in HCC. (**A**) Schematic outline of high-density CRISPR gene body scan in Cas9-expressing HepG2 and U87 cells. (**B** to **F**) Two-dimensional annotation of CRISPR gene tiling scans for (B) ACTR5, (C) INO80, (D) MCRS1, (E) ACTR8, and (F) YY1. The solid lines indicate the smoothened model of the CRISPR scan score derived from individual sgRNAs (dots) screened in HepG2 (red) and U87 (blue) cells. The median CRISPR scan scores of the positive control (dotted line; defined as −1.0) and negative control (defined as 0.0) sgRNAs are designated. (**G**) Three-dimensional annotation of ACTR5 CRISPR scan score relative to the AlphaFold structural model of ACTR5 (ID, Q9H9F9). (**H**) Effect of wild-type (WT)- and ΔA5-ACTR5 expression on the growth competition assay of HepG2-dCas9-Krab cells transduced with sgiACTR5 (n = 3 each group). (**I**) Silver stain of the TST-purified WT- and ΔA5-ACTR5 protein complexes. The top candidate of each protein band was suggested by LC-MS/MS. (**J**) Western blot of ACTR5 and IES6 in the TST-purified WT- and ΔA5-ACTR5 protein complexes. *P < 0.01 two-sided Student's t test.

to be exposed on the ACTR5 protein surface (Fig. 4G). Furthermore, the deletion of the A5 region blocked the capacity of ACTR5 to maintain HepG2 proliferation (Fig. 4H), indicating an indispensable role of this surface area of ACTR5 in HCC. To investigate the mechanisms of the A5 region in HCC, we expressed the TST-tagged wild-type (WT) and A5-deleted (ΔA5) ACTR5 in HepG2 cells and captured the ACTR5-containing complexes by Strep-Tactin XT beads. Characterization of the ACTR5-associated proteins using MS (LC-MS/MS) revealed a unique loss of interaction between ΔA5-ACTR5 and IES6 in HepG2 cells (Fig. 4I and data S11), which was readily confirmed by coimmunoprecipitation and Western blotting (Fig. 4J).

Stabilization of ACTR5 via interacting with IES6 modulates CDK inhibitory therapy

To examine the role of IES6 in HCC, we transduced the HepG2 cells with CRISPRi sgRNAs targeting *IES6* (sgiIES6) and found that cells expressing sgiIES6 were outcompeted compared to cells transduced with sgiNTC (Fig. 5A). We then sought to characterize the domains in IES6 that mediate the interaction with ACTR5. Similar to the ACTR5 gene body scan (shown in Fig. 4, A and B), we developed an IES6 CRISPR gene body scan library and screened in Cas9-expressing HepG2 and U87 cells. These parallel gene body scans revealed two HCC-selective essential regions, I1 (D124-P138) and I2 (T163-V187) in IES6 (Fig. 5B and data S12). AlphaFold modeling of IES6 indicated a structured C terminus, which contains the I2 element (Fig. 5C). On the other hand, the I1 region was predicted as an unfolded linker. Expression of the TST-tagged WT-, ΔI1-, and ΔI2-IES6 in HepG2 cells revealed loss of interaction to ACTR5 only in the ΔI2-IES6 condition (Fig. 5D). Furthermore, the deletion of the I2 region blocked the capacity of IES6 to maintain the HepG2 proliferation (Fig. 5E). Additionally, we found that CRISPRi depletion of IES6 reduced the protein level of ACTR5 (Fig. 5F). This phenomenon was also observed in the cells transduced with ΔI2-IES6 (Fig. 5D), indicating the role of IES6 in stabilizing the ACTR5 protein via protein-protein interaction. CRISPRi of IES6 also induced CDKN2A expression and inhibited the CDK6/p-Rb/E2F1 axis (Fig. 5F), recapitulating the cell cycle blockade triggered by the loss of ACTR5 (Fig. 3A). Therefore, our results point to a collaborative mechanism between ACTR5 and IES6 in supporting the cell cycle progression and cell proliferation in HCC. These observations also suggested that further inhibition of the CDK6 activity might exert a more pronounced cellular suppression in the ACTR5 (or IES6)–depleted HCC. Simultaneously targeting ACTR5/IES6 (by CRISPRi) and CDK6 (by ribociclib, a U.S. Food and Drug Administration–approved CDK4/6 inhibitor also known as LEE011 and KISQALI) (*39*) synergistically suppressed the HepG2 cell growth (Fig. 5, G and H), highlighting the possible combinatorial targeting of the ACTR5/IES6 and CDK6 axis for advanced HCC treatment (Fig. 5I).

DISCUSSION

HCC is one of the leading causes of cancer-related mortality worldwide (*1*, *2*). In this study, we performed multiomics analyses, including functional genomics (i.e., CRISPRi and high-density

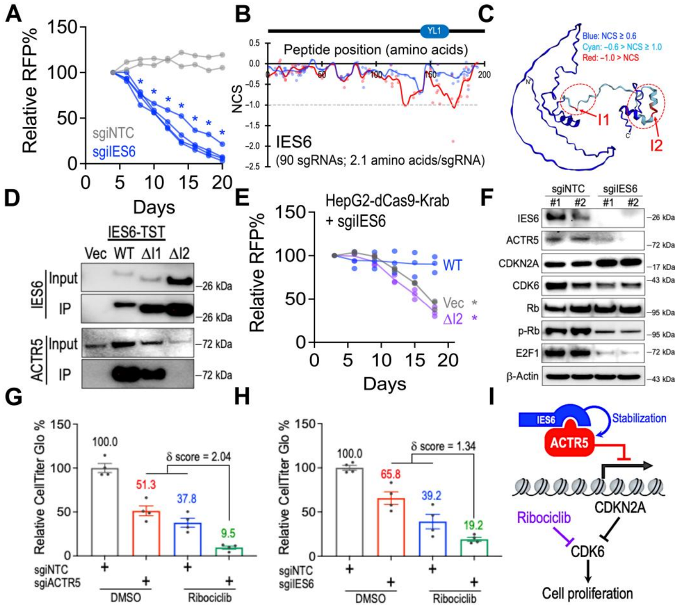

Fig. 5. IES6 stabilizes ACTR5 to support CDK6 signaling in HCC. (**A**) Growth competition assay of HepG2-dCas9-Krab cells transduced with RFP-labeled nontargeting control (gray lines; $n = 2$ independent sgiNTC sequences) and *IES6*-targeting sgiRNAs (blue lines; $n = 4$ independent sgiIES6 sequences). (**B**) Two-dimensional annotation of CRISPR gene tiling scans for IES6 in HepG2 (red) and U87 (blue) cells. The solid lines indicate the smoothened model of the CRISPR scan score derived from individual sgRNAs (dots). The median CRISPR scan scores of the positive control (dotted line; defined as −1.0) and negative control (defined as 0.0) sgRNAs are designated. (**C**) Three-dimensional annotation of IES6 CRISPR scan score relative to the AlphaFold structural model of IES6 (ID, Q6PI98). (**D**) Western blot of IES6 and ACTR5 in the TST-purified WT-, ΔI1-, and ΔI2-IES6 protein complexes. (**E**) Effect of WT- and ΔI2-IES6 expression on the growth competition assay of HepG2-dCas9-Krab cells transduced with sgiIES6 ($n = 3$ each group). (**F**) Western blot of IES6, ACTR5, CDKN2A, CDK6, Rb, p-Rb, E2F1, and β-actin in HepG2-dCas9-Krab cells transduced with sgiNTC and sgiIES6. (**G** and **H**) CellTiter-Glo analysis of the (G) sgiACTR5- and (H) sgiIES6-transduced HepG2-dCas9-Krab cells incubated with dimethyl sulfoxide (DMSO) or ribociclib ($n = 4$ each group). (**I**) Model of the ACTR5/IES6 complex supporting CDK6-driven cell proliferation. Data are presented as means ± SEM. $^*P < 0.01$ by two-sided Student's *t* test.

CRISPR gene body screens), transcriptomics (RNA-seq), epigenetics (ChIP-seq), and proteomics (MS) in the HCC cells. Using these systems biological approaches, we identified a critical role of the ACTR5 in HCC disease progression. We further demonstrate that ACTR5 contributes to cell cycle progression via suppressing CDKN2A, which can be exploited to enhance the efficacy of CDK6-targeted therapies.

Genome-wide CRISPR-KO screens have been performed in more than 1000 cell lines (DepMap Project; including 22 HCC lines) (*19*, *20*) and identified numerous insights underlying the liver malignancies (*40*–*42*). Unexpectedly, these large-scale screen efforts did not reveal the essential role of ACTR5 in HCC (Fig. 1C). To this end, we aligned the targeting positions of sgACTR5 used in the DepMap genome-wide CRISPR library to our ACTR5 gene tiling scan (fig. S8A; blue dots). We noted that three of the four sgACTR5 in the DepMap screens indeed missed the CRISPR-sensitive regions (NCS < −1.0) of ACTR5, causing a false-negative assessment of this HCC essential gene within the consortium databases (a similar issue was also noted for IES6 in the DepMap database; fig. S8B). In contrast, our epigenetic-focused CRISPRi library screen acted through suppression of target gene expression successfully captured the loss-of-function phenotype in the ACTR5-depleted HCC cells (Fig. 1, A and B). Furthermore, our CRISPR gene tiling approach that used all possible sgRNAs (i.e., 254 sgRNAs tiling ACTR5) to probe the gene coding regions provided an additional layer of domain resolution (Fig. 4, A and B). Collectively, our study demonstrated the utility of serial and focused CRISPR genetic screens in finding additional cancer essential genes over the conventional genome-wide CRISPR screens.

ACTR5 is commonly considered a core component of the INO80 chromatin remodeling complex, which has been implicated in adenosine triphosphate (ATP)–dependent nucleosome remodeling, DNA repair, and transcriptional regulation (*43*–*46*). The INO80 complex members are highly conserved from primitive eukaryotic organisms to mammals, highlighting its crucial roles in fundamental chromatin biology (*47*). Structural-based analysis revealed that the ACTR5/IES6 module is vital for optimizing the nucleosome binding of the INO80 complex (*24*, *25*, *48*, *49*). Our parallel gene body scan of the critical components of the INO80 complex revealed a unique HepG2-specific CRISPR sensitivity in the ACTR5/IES6 coding regions (i.e., A1 to A6 and I1 and I2 regions observed in Figs. 4B and 5B) as compared to the other INO80 members (Fig. 4, C to F; no HepG2-specific area was observed in INO80, MCRS1, ACTR8, and YY1). Furthermore, we detected a clear interaction between ACTR5 and IES6 via MS and coimmunoprecipitation immunoblotting (Fig. 4, I and J); however, the other components of the INO80 complex were not readily observed in the ACTR5-associated protein pool (data S11). These results support the hypothesis that ACTR5 and IES6 predominantly assembled as an independent subcomplex with a distinct function from the conventional INO80 complex. Our epigenomic data revealed that 21.1% (111 of 525) of ACTR5-targeted genes was not recognized by the canonical INO80 complex (fig. S9; including *CDKN2A*) (*50*). Furthermore, 27.0% of these ACTR5-specific target genes exerted increased mRNA levels upon CRISPRi of ACTR5, which is twofold more than in the INO80-bound genes (14.5%), highlighting a repressive role of ACTR5 on the target gene transcription (data S13). To this end, we found that ACTR5 modulates the level of H3K9me2 at the *CDKN2A* TSS locus in HCC (Fig. 2F and fig. S10A). Last, our correlation study suggests that the expression level of *CDKN2A* is associated with the cellular response to ACTR5 depletion (fig. S11A), supporting a central role of the ACTR5-CDKN2A axis in regulating the cell cycle and tumor progression. Of note, the derepression of CDKN2A (fig. S10B) and the inactivation of CDK6/p-Rb/E2F1 (fig. S10C) were also observed in MV411 cells (AML cells exhibited similar sensitivity to ACTR5 depletion as SNU475 cells in Fig. 1E), indicating that the ACTR5-directed regulation of CDKN2A may exist across multiple cancer types, extending the impact of the current study beyond HCC.

CDKN2A (also known as $P14^{ARF}$ or $P16^{INK4A}$) is a G_1-S cell cycle regulator and tumor repressor, the loss-of-function mutations and epigenetic inactivation of which have been observed in various cancer types, including HCC (*8*, *9*). CDKN2A inhibits CDK4/6 to prevent Rb phosphorylation, thus maintaining Rb's ability to block the E2F-driven cell cycle program (*15*, *16*). Given that CDKN2A silencing and CDK overexpression have been frequently observed in

patients with HCC, CDK inhibitors appear to be an attractive strategy for HCC treatment (*16*, *39*, *51*, *52*). We found that ACTR5 acts as the suppressor of CDKN2A, thereby supporting CDK6 signaling and cell proliferation. Consistently, our data revealed that a stronger ACTR5 dependency could be predicted by a shorter doubling time within the tested HCC cells (fig. S11B), suggesting that inhibiting ACTR5 may provide a more potent suppression of the fast proliferating (i.e., more malignant) cells. Targeting ACTR5 (and its complex partner IES6) thus represent viable therapeutic approaches for HCC treatment. Furthermore, the synergistic effect between ACTR5/IES6 suppression and CDK4/6 inhibition on HCC proliferation opens a possibility of combinational therapy against HCC (Fig. 5, G to I). Given that ribociclib (also known as LEE011 or KISQALI) is currently undergoing phase 1 and 2 clinical trials in patients with advanced HCC (NCT02524119) (*39*), our study provides an additional layer of mechanism and therapeutic opportunity to further improve the CDK-targeted therapy in HCC and beyond.

In summary, our study highlighted that ACTR5 is required for HCC proliferation via suppressing CDKN2A expression, which is independent of the conventional INO80 complex activity. Disruption of ACTR5 (or its complex partner IES6) synergizes with pharmacological targeting of CDK4/6, providing critical rationales toward a more effective combinatorial therapy against HCC and beyond. Furthermore, the insights into the roles of ACTR5/IES6 domain interactions may prompt future efforts to discover novel classes of molecules targeting this interface. Although the cell cycle regulator pathways are recognized to play pivotal roles in multiple cancer types, studies on CDK-targeted therapy have focused primarily on inhibiting a single kinase to suppress cell proliferation. The dynamic interplays between the CDK activation network and the therapeutic outcome are just beginning to gain recognition. This study thus represents one of the emerging research fields that explore how the epigenetic effectors coordinate in a broad spectrum of biological processes, such as gene transcription, cell cycle signaling, and therapeutic efficacy.

MATERIALS AND METHODS

Cell culture

HepG2 [HB-8065, American Type Culture Collection (ATCC)], U87 (HTB-14, ATCC), U251, MDA-MB-231 (CRM-HTB-26, ATCC), MCF7 (HTB-22, ATCC), and human embryonic kidney (HEK) 293 (CRL-1573, ATCC) cells were cultured in Dulbecco's modified Eagle's medium (Gibco) supplemented with 10% fetal bovine serum (FBS) (Omega Scientific). MV4-11 (CRL-9591, ATCC), MOLM13, SNU182 (CRL-2235, ATCC), and SNU475 (CRL-2236, ATCC) were cultured in RPMI 1640 (Gibco) with 10% FBS. Penicillin-streptomycin (Gibco) and plasmocin (0.5 μg/ml; Invitrogen) were added to all media. All cells were cultured in a 37°C incubator with 5% CO_2. Cells stably expressing the Cas9 or dCas9-Krab were established via transduction of LentiCas9-blast (52962, Addgene) or LentidCas9-Krab-blast (89567, Addgene) lentivirus and selected by blasticidin (20 μg/ml; Gibco), single-cell cloning, and CRISPR efficiency test (fig. S2). The response of sgiNTC-, sgiACTR5-, and sgiIES6-transduced HepG2-dCas9-Krab cells to ribociclib (HY-15777, Medchemexpress) was measured by CellTiter-Glo 2.0 reagent (G9242, Promega).

Lentiviral CRISPR library and cDNA construction

For the epigenetics-focused CRISPRi library, 3669 sgiRNA sequences targeting the TSS of 729 epigenetic-related genes were designed using the human genome-wide CRISPRi-v2 (*53*). For the INO80 complex member gene body scan CRISPR libraries, sgRNA sequences targeting the coding exons of the select genes (*ACTR5*, *IES6*, *INO80*, *MCRS1*, *ACTR8*, and *YY1*) were designed using the Genetic Perturbation Platform (Broad Institute) (*54*). Briefly, guide RNA oligos were synthesized by microarray (CustomArray) and cloned into the ipUSEPR lentiviral sgRNA vector (hU6-driven sgRNA coexpressed with EF-1a–driven RFP and puromycin-resistance gene) using the Bsm BI (NEB) restriction sites (*22*, *23*, *55*) (fig. S1A). Individual sgRNAs selected for validation experiments are listed in tables S1 and S2. The cDNA of WT ACTR5 and IES6 that fused with a TST were designed using the CLC Main Workbench (Qiagen), synthesized by gBlock gene fragments (IDT), and cloned into the lentiviral pLVN vector (EF-1a–driven transgene coexpressed with neomycin-resistance gene) using the NEBuilder HiFi DNA assembly cloning kit (NEB). The final plasmids were validated via Sanger sequencing (Eton Bioscience). All molecular cloning was performed using the NEB 5-alpha Competent *Escherichia coli* (C2987H; NEB). Lentivirus was produced in HEK293 cells (CRL-1573, ATCC) by cotransfecting ipUSEPR or pLVN vectors with the packaging plasmids pPAX2 (12260, Addgene) and pMD2.G (12259, Addgene). For lentiviral infection, target cells were mixed with the viral solution and polybrene (8 μg/ml; TR1003G, Millipore Sigma) and incubated overnight.

CRISPR library screen and analysis

The epigenetic-focused CRISPR library was delivered into HepG2-dCas9-Krab cells (for CRISPRi), and the INO80 complex member gene scan CRISPR library was delivered into HepG2-Cas9 and U87-Cas9 cells (for CRISPR KO) (fig. S2). Briefly, cells were infected with the CRISPR library at ~15% infection (monitored by flow cytometry for RFP expression; three replicates each screen) and selected by puromycin (2 μg/ml; Gibco). The library-transduced cells were subcultured every 4 days for a total of 24 days. At the start (day 0) and end (day 24) time points, 4 million cells from each screen culture were collected. The integrated sgRNA in each sample was PCR-amplified (NEBNext Ultra II Q5, NEB) using primers DCF01 5′-CTTGTGGAAAGGACGAAACACCG-3′ and DCR03 5′-CCTAGGAACAGCGGTTTAAAAAAGC-3′ for high-throughput sequencing (NextSeq550, Illumina). To quantify sgRNA reads, 20-nucleotide sequences that matched the sgRNA backbone structure (5′-CACCG and GTTT-3′) were extracted and mapped to the library sgRNA sequences using Bowtie2. The frequency for individual sgRNAs was calculated as the read counts of each sgRNA divided by the total read counts matched to the library. For the epigenetics-focused CRISPRi screen, the top essential candidate genes were analyzed using the MAGeCK algorithm (*26*). For the INO80 complex member gene scan, the NCS was defined as a $\log_{10}$-fold change in the frequency of individual sgRNAs between the start (day 0) and end (day 24) of the screened samples and normalized by the median score of the negative control sgRNA (defined as 0.0; sgRNA targeting nonessential sequences) and the median score of the positive control sgRNA (defined as −1.0; sgRNA targeting *MYC*, *BRD4*, *RPA3*, *PCNA*, etc.) within the screen data. The underrepresented sgRNAs (less than 5% of the average frequency) in the library were excluded from the

analysis. The NCS of individual sgRNA was processed by Gaussian kernel smoothing in R, and the average score over the trinucleotide codons was calculated for each peptide position. Next, three-dimensional structural models of ACTR5 (Q9H9F9) and IES6 (Q6PI98) were obtained from the AlphaFold database (*38*). Subsequently, the smoothened NCSs were mapped onto three-dimensional structures using the "Defined Attribute" and "Render by Attribute" functionalities in UCSF Chimera 1.15.

Flow cytometric assays

For competition cell culture assays, Cas9- or dCas9-Krab–expressing cells were transduced with the ipUSEPR sgRNA (RFP-positive) constructs in 96-well plates at ~50% infection. The cell viability and the percentage of RFP-positive were obtained by high-throughput flow cytometry and 4′,6-diamidino-2-phenylindole (Invitrogen) dye exclusion. Cell cycle was monitored by Click-iT Plus EdU Alexa Fluor 647 assay kits (C10634, Invitrogen). Data were obtained by high-throughput flow cytometry using an Attune NxT flow cytometer with an autosampler (Thermo Fisher Scientific).

Western blotting

Cells were harvested and lysed in lithium dodecyl sulfate sample buffer (Invitrogen) at 5×10^6 cells/ml, separated electrophoretically using Bolt 4 to 12% bis-tris plus gels (Invitrogen), and transferred onto polyvinylidene difluoride (PVDF) membranes (0.2 μm pore size) using PVDF Mini Stacks and iBlot 2 (Invitrogen). Membranes were immersed in 5% nonfat milk then probed with rabbit antibodies against ACTR5 (sc-376364, Santa Cruz Biotechnology; 1:1000), IES6 (PA5-61869, Thermo Fisher Scientific; 1:1000), CDKN2A (ab108349, Abcam; 1:1000), CDK6 (ab124821, Abcam; 1:1000), E2F1 (3742S, Cell Signaling Technology; 1:1000), Rb (ab181616, Abcam; 1:1000), phospho-S780 Rb (ab173289, Abcam; 1:1000), and β-actin (4970S, Cell Signaling Technology; 1:1000) at 4°C overnight. After washing, the membranes were incubated with horseradish peroxidase–linked goat anti-rabbit immunoglobulin G antibody (31460, Invitrogen; 1: 200,000) at room temperature for 1 hour. Chemiluminescent signals were developed using the SuperSignal West Femto Substrate (Thermo Fisher Scientific) and detected using a ChemiDoc imaging system (Bio-Rad).

Mass spectrometry

The cell lysates harvested from pLVN-vector and pLVN-ACTR5-TST (WT and ΔA5)–transduced HepG2 cells were incubated with MagStrep "type3" XT Beads (2-4090-002, IBA; 1:1000) at 4°C for 30 min. The beads were washed three times in 1× Buffer W (2-1003-100, IBA), and the TST-captured proteins were eluted by 1× Buffer BXT (2-1042-025, IBA; containing biotin). For silver stain, the TST-captured proteins were first separated by a Bolt 4 to 12% bis-tris plus gels (Invitrogen). The electrophoresis gels were fixed in 0.02% formaldehyde (Sigma-Aldrich) and stained with 0.1% silver nitrate (Sigma-Aldrich). The signals were developed with 6% sodium carbonate (Thermo Fisher Scientific). Once desired intensity was obtained, the reaction was stopped by incubating with 12% acetic acid (Thermo Fisher Scientific). Silver staining was detected using a ChemiDoc imaging system (Bio-Rad). For MS, the gel regions containing the indicated protein bands were destained with potassium ferricyanide and sodium thiosulfate, reduced with 10 mM dithiothreitol, alkylated with 55 mM iodoacetamide, and digested with trypsin/LysC (*56*). The digested peptides were extracted from the gel, dried, and resuspended in water with 2% acetonitrile and 0.1% formic acid. Peptides were then desalted using ZipTips (EMD Millipore) and resuspended in 2% acetonitrile with 0.1% formic acid. Each digest was analyzed by LC-MS/MS on an Orbitrap Lumos mass spectrometer at the Integrated Mass Spectrometry Shared Resource of the City of Hope Comprehensive Cancer Center. Among the top 20 abundant peptides observed at each protein band area, the protein that exhibits the highest enrichment ratio between the ACTR5-TST (WT) and vector samples was annotated (Fig. 4I and data S11).

ACTR5-associated genomic DNA sequencing

For detecting the ACTR5-targeted genomic regions, HepG2 cells expressing pLVN vector or pLVN-ACTR5-TST were incubated with 1% (v/v) formaldehyde at room temperature for 10 min, followed by the addition of 125 mM glycine to quench the excessive formaldehyde. The fixed cells were lysed in ChIP SDS lysis buffer [1% SDS, 10 mM EDTA, and 50 mM tris-HCl (pH 8.0)] supplemented with Halt Protease Inhibitor Cocktail (78430, Thermo Fisher Scientific), and the chromatin was fragmented by Bioruptor sonication (Diagenode). The sheared chromatin sample was then incubated with the MagStrep "type3" XT Beads (2-4090-002, IBA) and washed with a low-salt buffer [0.1% SDS, 1% Triton X-100, 2 mM EDTA, 150 mM NaCl, and 20 mM tris-HCl (pH 8.0)], followed by a high-salt buffer [0.1% SDS, 1% Triton X-100, 2 mM EDTA, 500 mM NaCl, and 20 mM tris-HCl (pH 8.0)], a LiCl wash buffer [250 mM LiCl, 1% IGEPAL-CA630, 1% deoxycholic acid, 1 mM EDTA, and 10 mM tris-HCl (pH 8.0)], and the TE buffer [1 mM EDTA and 10 mM tris-HCl (pH 8.0)]. The washed beads were then incubated with reverse cross-linking buffer (1.1% SDS and 110 mM sodium bicarbonate) at 65°C overnight, followed by GeneJET DNA purification (K0702, Thermo Fisher Scientific), and the enriched genomic DNA was submitted for library prep and NovaSeq6000 sequencing (Novogene). The raw sequence reads were quality checked using the FASTQC software (version 0.11.8) and aligned against the human genome hg38 using Burrows-Wheeler Aligner (version 0.7.17). The aligned reads were then sorted by Samtools (version 1.10), and the duplicated reads were removed by Picard MarkDuplicates (version 2.21.1). Peak-calling analysis to identify antibody-binding regions was performed using MACS2 (version 2.1.1), and the SPMR option was used to generate normalized pileup files for downstream analysis. ChIP-seq signals were calculated from the pileup files around TSS ± 1-kb regions and visualized in plots using deepTools (version 3.3.0). The sequencing results were validated by real-time qPCR (primers listed in table S3) using the PowerUp SYBR Green Master Mix (Thermo Fisher Scientific) and a QuantStudio 3 Real-Time PCR System (Applied Biosystems).

Transcriptomic analysis

For RNA-seq, total RNA was extracted using the RNeasy Mini Kit (74104, QIAGEN) and submitted for mRNA library prep and NovaSeq6000 sequencing (Novogene). Raw sequence reads were mapped to the human genome (GRCh38) using STAR v2.5.3 and calculated using featureCounts v1.5.1. The raw counts were then normalized using the trimmed mean of M values method and compared using the Bioconductor package "edgeR." Genes with a minimum average of one read per kilobase per million were selected for analysis. GSEA was performed using the GSEA v4.1.0 (University of California, San Diego and Broad Institute).

HepG2 xenograft modeling

NSG (NOD scid gamma; NOD.Cg-*Prkdc*^{scid} *Il2rg*^{tm1Wjl}/SzJ) mice were housed at the animal core facility of City of Hope and used to generate the HepG2 xenograft model. NSG mice (6 to 8 weeks old) were randomly assigned to experimental groups. One million HepG2 cells transduced with sgiNTC or sgiACTR5 were resuspended in 100 μl of phosphate-buffered saline and mixed at a 1:1 ratio with Matrigel matrix (356237, Corning) for subcutaneous injection in the NSG mice (four tumor sites per mouse). Mice were euthanized at 24 days after transplantation, and the tumor tissues were collected. All the mouse experiments were approved by the Institutional Animal Care and Use Committee at City of Hope Comprehensive Cancer Center.

Code availability

The computational codes/tool packages used in this study include Genetic Perturbation Platform (Broad Institute), Bowtie2 (Johns Hopkins University), MAGeCK (Dana-Farber Cancer Institute), UCSF Chimera 1.15 (University of California, San Francisco), Attune NxT v3.1.2 (Thermo Fisher Scientific), GSEA v4.1.0 (University of California, San Diego and Broad Institute), FASTQC v0.11.8, MACS2 v2.1.1, STAR v2.5.3, featureCounts v1.5.1, edgeR, IGV 2.11.0 (Broad Institute), BioRender (https://biorender.com), QuantStudio Design and Analysis Software v1.5.1 (Applied Biosystems), Bio-Rad ChemiDoc MP (Bio-Rad), and DAVID (https://david.ncifcrf.gov/). Two-sided Student's *t* tests were carried out using Prism 9 (GraphPad) to determine the statistical significance of difference between variables.

Supplementary Materials

This PDF file includes:
Figs. S1 to S11
Tables S1 to S3

Other Supplementary Material for this manuscript includes the following:
Suppl excel data table

View/request a protocol for this paper from *Bio-protocol*.

REFERENCES AND NOTES

1. H. Sung, J. Ferlay, R. L. Siegel, M. Laversanne, I. Soerjomataram, A. Jemal, F. Bray, Global Cancer Statistics 2020: GLOBOCAN estimates of incidence and mortality worldwide for 36 cancers in 185 countries. *CA Cancer J. Clin.* **71**, 209–249 (2021).
2. A. Villanueva, Hepatocellular carcinoma. *N. Engl. J. Med.* **380**, 1450–1462 (2019).
3. A. Forner, J. M. Llovet, J. Bruix, Hepatocellular carcinoma. *Lancet* **379**, 1245–1255 (2012).
4. J. M. Llovet, S. Ricci, V. Mazzaferro, P. Hilgard, E. Gane, J.-F. Blanc, A. C. de Oliveira, A. Santoro, J.-L. Raoul, A. Forner, M. Schwartz, C. Porta, S. Zeuzem, L. Bolondi, T. F. Greten, P. R. Galle, J.-F. Seitz, I. Borbath, D. Häussinger, T. Giannaris, M. Shan, M. Moscovici, D. Voliotis, J. Bruix; SHARP Investigators Study Group, Sorafenib in advanced hepatocellular carcinoma. *N. Engl. J. Med.* **359**, 378–390 (2008).
5. G. G. Wang, C. D. Allis, P. Chi, Chromatin remodeling and cancer, Part II: ATP-dependent chromatin remodeling. *Trends Mol. Med.* **13**, 373–380 (2007).
6. I. Anestopoulos, G. P. Voulgaridou, A. G. Georgakilas, R. Franco, A. Pappa, M. I. Panayiotidis, Epigenetic therapy as a novel approach in hepatocellular carcinoma. *Pharmacol. Ther.* **145**, 103–119 (2015).
7. T. Hardy, D. A. Mann, Epigenetics in liver disease: From biology to therapeutics. *Gut* **65**, 1895–1905 (2016).
8. The Cancer Genome Atlas Research Network, D. A. Wheeler, L. R. Roberts, Comprehensive and integrative genomic characterization of hepatocellular carcinoma. *Cell* **169**, 1327–1341.e23 (2017).
9. J. M. Llovet, J. Zucman-Rossi, E. Pikarsky, B. Sangro, M. Schwartz, M. Sherman, G. Gores, Hepatocellular carcinoma. *Nat. Rev. Dis. Primers* **2**, 16018 (2016).
10. K. Schulze, S. Imbeaud, E. Letouze, L. B. Alexandrov, J. Calderaro, S. Rebouissou, G. Couchy, C. Meiller, J. Shinde, F. Soysouvanh, A.-L. Calatayud, R. Pinyol, L. Pelletier, C. Balabaud, A. Laurent, J.-F. Blanc, V. Mazzaferro, F. Calvo, A. Villanueva, J. C. Nault, P. Bioulac-Sage, M. R. Stratton, J. M. Llovet, J. Zucman-Rossi, Exome sequencing of hepatocellular carcinomas identifies new mutational signatures and potential therapeutic targets. *Nat. Genet.* **47**, 505–511 (2015).
11. D.-Y. Wang, L.-P. Zou, X.-J. Liu, H.-G. Zhu, R. Zhu, Hepatitis B virus X protein induces the histone H3 lysine 9 trimethylation on the promoter of p16 gene in hepatocarcinogenesis. *Exp. Mol. Pathol.* **99**, 399–408 (2015).
12. M. Arechederra, M. Recalde, M. Gárate-Rascón, M. G. Fernández-Barrena, M. A. Ávila, C. Berasain, Epigenetic biomarkers for the diagnosis and treatment of liver disease. *Cancers (Basel)* **13**, 1265 (2021).
13. Y. Kondo, L. Shen, S. Suzuki, T. Kurokawa, K. Masuko, Y. Tanaka, H. Kato, Y. Mizuno, M. Yokoe, F. Sugauchi, N. Hirashima, E. Orito, H. Osada, R. Ueda, Y. Guo, X. Chen, J. P. Issa, Y. Sekido, Alterations of DNA methylation and histone modifications contribute to gene silencing in hepatocellular carcinomas. *Hepatol. Res.* **37**, 974–983 (2007).
14. C. T. Liew, H. M. Li, K. W. Lo, C. K. Leow, J. Y. Chan, L. Y. Hin, W. Y. Lau, P. B. Lai, B. K. Lim, J. Huang, W. T. Leung, S. Wu, J. C. Lee, High frequency of p16INK4A gene alterations in hepatocellular carcinoma. *Oncogene* **18**, 789–795 (1999).
15. X. Bisteau, M. J. Caldez, P. Kaldis, The complex relationship between liver cancer and the cell cycle: A story of multiple regulations. *Cancers (Basel)* **6**, 79–111 (2014).
16. M. Malumbres, M. Barbacid, Cell cycle, CDKs and cancer: A changing paradigm. *Nat. Rev. Cancer* **9**, 153–166 (2009).
17. L. A. Gilbert, M. A. Horlbeck, B. Adamson, J. E. Villalta, Y. Chen, E. H. Whitehead, C. Guimaraes, B. Panning, H. L. Ploegh, M. C. Bassik, L. S. Qi, M. Kampmann, J. S. Weissman, Genome-scale CRISPR-mediated control of gene repression and activation. *Cell* **159**, 647–661 (2014).
18. X. Shen, R. Ranallo, E. Choi, C. Wu, Involvement of actin-related proteins in ATP-dependent chromatin remodeling. *Mol. Cell* **12**, 147–155 (2003).
19. A. Tsherniak, F. Vazquez, P. G. Montgomery, B. A. Weir, G. Kryukov, G. S. Cowley, S. Gill, W. F. Harrington, S. Pantel, J. M. Krill-Burger, R. M. Meyers, L. Ali, A. Goodale, Y. Lee, G. Jiang, J. Hsiao, W. F. J. Gerath, S. Howell, E. Merkel, M. Ghandi, L. A. Garraway, D. E. Root, T. R. Golub, J. S. Boehm, W. C. Hahn, Defining a cancer dependency map. *Cell* **170**, 564–576.e16 (2017).
20. R. M. Meyers, J. G. Bryan, J. M. McFarland, B. A. Weir, A. E. Sizemore, H. Xu, N. V. Dharia, P. G. Montgomery, G. S. Cowley, S. Pantel, A. Goodale, Y. Lee, L. D. Ali, G. Jiang, R. Lubonja, W. F. Harrington, M. Strickland, T. Wu, D. C. Hawes, V. A. Zhivich, M. R. Wyatt, Z. Kalani, J. J. Chang, M. Okamoto, K. Stegmaier, T. R. Golub, J. S. Boehm, F. Vazquez, D. E. Root, W. C. Hahn, A. Tsherniak, Computational correction of copy number effect improves specificity of CRISPR-Cas9 essentiality screens in cancer cells. *Nat. Genet.* **49**, 1779–1784 (2017).
21. J. Shi, E. Wang, J. P. Milazzo, Z. Wang, J. B. Kinney, C. R. Vakoc, Discovery of cancer drug targets by CRISPR-Cas9 screening of protein domains. *Nat. Biotechnol.* **33**, 661–667 (2015).
22. L. Yang, A. K. N. Chan, K. Miyashita, C. D. Delaney, X. Wang, H. Li, S. P. Pokharel, S. Li, M. Li, X. Xu, W. Lu, Q. Liu, N. Mattson, K. Y. Chen, J. Wang, Y.-C. Yuan, D. Horne, S. T. Rosen, Y. Soto-Feliciano, Z. Feng, T. Hoshii, G. Xiao, M. Müschen, J. Chen, S. A. Armstrong, C.-W. Chen, High-resolution characterization of gene function using single-cell CRISPR tiling screen. *Nat. Commun.* **12**, 4063 (2021).
23. Q. Liu, A. K. N. Chan, W.-H. Chang, L. Yang, S. P. Pokharel, K. Miyashita, N. Mattson, X. Xu, M. Li, W. Lu, R.-J. Lin, S.-Y. Wang, C.-W. Chen, 3-Ketodihydrosphingosine reductase maintains ER homeostasis and unfolded protein response in leukemia. *Leukemia* **36**, 100–110 (2022).
24. S. Eustermann, K. Schall, D. Kostrewa, K. Lakomek, M. Strauss, M. Moldt, K.-P. Hopfner, Structural basis for ATP-dependent chromatin remodelling by the INO80 complex. *Nature* **556**, 386–390 (2018).
25. R. Ayala, O. Willhoft, R. J. Aramayo, M. Wilkinson, E. A. McCormack, L. Ocloo, D. B. Wigley, X. Zhang, Structure and regulation of the human INO80-nucleosome complex. *Nature* **556**, 391–395 (2018).
26. W. Li, H. Xu, T. Xiao, L. Cong, M. I. Love, F. Zhang, R. A. Irizarry, J. S. Liu, M. Brown, X. S. Liu, MAGeCK enables robust identification of essential genes from genome-scale CRISPR/Cas9 knockout screens. *Genome Biol.* **15**, 554 (2014).
27. Z. Tang, C. Li, B. Kang, G. Gao, C. Li, Z. Zhang, GEPIA: A web server for cancer and normal gene expression profiling and interactive analyses. *Nucleic Acids Res.* **45**, W98–W102 (2017).
28. A. Subramanian, P. Tamayo, V. K. Mootha, S. Mukherjee, B. L. Ebert, M. A. Gillette, A. Paulovich, S. L. Pomeroy, T. R. Golub, E. S. Lander, J. P. Mesirov, Gene set enrichment analysis: A knowledge-based approach for interpreting genome-wide expression profiles. *Proc. Natl. Acad. Sci. U.S.A.* **102**, 15545–15550 (2005).

29. T. G. Schmidt, L. Batz, L. Bonet, U. Carl, G. Holzapfel, K. Kiem, K. Matulewicz, D. Niermeier, I. Schuchardt, K. Stanar, Development of the Twin-Strep-tag® and its application for purification of recombinant proteins from cell culture supernatants. *Protein Expr. Purif.* **92**, 54–61 (2013).
30. ENCODE Project Consortium, An integrated encyclopedia of DNA elements in the human genome. *Nature* **489**, 57–74 (2012).
31. K. E. Bachman, B. H. Park, I. Rhee, H. Rajagopalan, J. G. Herman, S. B. Baylin, K. W. Kinzler, B. Vogelstein, Histone modifications and silencing prior to DNA methylation of a tumor suppressor gene. *Cancer Cell* **3**, 89–95 (2003).
32. K. Agger, P. A. Cloos, L. Rudkjaer, K. Williams, G. Andersen, J. Christensen, K. Helin, The H3K27me3 demethylase JMJD3 contributes to the activation of the *INK4A-ARF* locus in response to oncogene- and stress-induced senescence. *Genes Dev.* **23**, 1171–1176 (2009).
33. S. Ortega, M. Malumbres, M. Barbacid, Cyclin D-dependent kinases, INK4 inhibitors and cancer. *Biochim. Biophys. Acta* **1602**, 73–87 (2002).
34. U. Raudvere, L. Kolberg, I. Kuzmin, T. Arak, P. Adler, H. Peterson, J. Vilo, g:Profiler: A web server for functional enrichment analysis and conversions of gene lists (2019 update). *Nucleic Acids Res.* **47**, W191–W198 (2019).
35. A. Torsvik, D. Stieber, P. Ø. Enger, A. Golebiewska, A. Molven, A. Svendsen, B. Westermark, S. P. Niclou, T. K. Olsen, M. Chekenya Enger, R. Bjerkvig, U-251 revisited: Genetic drift and phenotypic consequences of long-term cultures of glioblastoma cells. *Cancer Med.* **3**, 812–824 (2014).
36. M. J. Clark, N. Homer, B. D. O'Connor, Z. Chen, A. Eskin, H. Lee, B. Merriman, S. F. Nelson, U87MG decoded: The genomic sequence of a cytogenetically aberrant human cancer cell line. *PLOS Genet.* **6**, e1000832 (2010).
37. V. A. C. Schoonenberg, M. A. Cole, Q. Yao, C. Macias-Trevino, F. Sher, P. G. Schupp, M. C. Canver, T. Maeda, L. Pinello, D. E. Bauer, CRISPRO: Identification of functional protein coding sequences based on genome editing dense mutagenesis. *Genome Biol.* **19**, 169 (2018).
38. J. Jumper, R. Evans, A. Pritzel, T. Green, M. Figurnov, O. Ronneberger, K. Tunyasuvunakool, R. Bates, A. Žídek, A. Potapenko, A. Bridgland, C. Meyer, S. A. A. Kohl, A. J. Ballard, A. Cowie, B. Romera-Paredes, S. Nikolov, R. Jain, J. Adler, T. Back, S. Petersen, D. Reiman, E. Clancy, M. Zielinski, M. Steinegger, M. Pacholska, T. Berghammer, S. Bodenstein, D. Silver, O. Vinyals, A. W. Senior, K. Kavukcuoglu, P. Kohli, D. Hassabis, Highly accurate protein structure prediction with AlphaFold. *Nature* **596**, 583–589 (2021).
39. D. Tripathy, A. Bardia, W. R. Sellers, Ribociclib (LEE011): Mechanism of action and clinical impact of this selective cyclin-dependent kinase 4/6 inhibitor in various solid tumors. *Clin. Cancer Res.* **23**, 3251–3262 (2017).
40. L. Wei, D. Lee, C.-T. Law, M. S. Zhang, J. Shen, D. W.-C. Chin, A. Zhang, F. H.-C. Tsang, C. L.-S. Wong, I. O.-L. Ng, C. C.-L. Wong, C.-M. Wong, Genome-wide CRISPR/Cas9 library screening identified PHGDH as a critical driver for Sorafenib resistance in HCC. *Nat. Commun.* **10**, 4681 (2019).
41. C.-Q. Song, Y. Li, H. Mou, J. Moore, A. Park, Y. Pomyen, S. Hough, Z. Kennedy, A. Fischer, H. Yin, D. G. Anderson, D. Conte Jr., L. Zender, X. W. Wang, S. Thorgeirsson, Z. Weng, W. Xue, Genome-wide CRISPR screen identifies regulators of mitogen-activated protein kinase as suppressors of liver tumors in mice. *Gastroenterology* **152**, 1161–1173.e1 (2017).
42. M. H.-R. Bao, C. Yang, A. P. W. Tse, L. Wei, D. Lee, M. S. Zhang, C. C. Goh, D. K.-C. Chiu, V. W.-H. Yuen, C.-T. Law, W.-C. Chin, N. N.-Q. Chui, B. P.-Y. Wong, C. Y.-K. Chan, I. O.-L. Ng, C. Y. S. Chung, C.-M. Wong, C. C.-L. Wong, Genome-wide CRISPR-Cas9 knockout library screening identified PTPMT1 in cardiolipin synthesis is crucial to survival in hypoxia in liver cancer. *Cell Rep.* **34**, 108676 (2021).
43. X. Shen, G. Mizuguchi, A. Hamiche, C. Wu, A chromatin remodelling complex involved in transcription and DNA processing. *Nature* **406**, 541–544 (2000).
44. A. J. Morrison, J. Highland, N. J. Krogan, A. Arbel-Eden, J. F. Greenblatt, J. E. Haber, X. Shen, INO80 and gamma-H2AX interaction links ATP-dependent chromatin remodeling to DNA damage repair. *Cell* **119**, 767–775 (2004).
45. H. van Attikum, O. Fritsch, B. Hohn, S. M. Gasser, Recruitment of the INO80 complex by H2A phosphorylation links ATP-dependent chromatin remodeling with DNA double-strand break repair. *Cell* **119**, 777–788 (2004).
46. K. Yen, V. Vinayachandran, B. F. Pugh, SWR-C and INO80 chromatin remodelers recognize nucleosome-free regions near +1 nucleosomes. *Cell* **154**, 1246–1256 (2013).
47. A. J. Morrison, X. Shen, Chromatin remodelling beyond transcription: The INO80 and SWR1 complexes. *Nat. Rev. Mol. Cell Biol.* **10**, 373–384 (2009).
48. L. Chen, R. C. Conaway, J. W. Conaway, Multiple modes of regulation of the human Ino80 SNF2 ATPase by subunits of the INO80 chromatin-remodeling complex. *Proc. Natl. Acad. Sci. U.S.A.* **110**, 20497–20502 (2013).
49. W. Yao, D. A. King, S. L. Beckwith, G. J. Gowans, K. Yen, C. Zhou, A. J. Morrison, The INO80 Complex requires the Arp5-Ies6 subcomplex for chromatin remodeling and metabolic regulation. *Mol. Cell. Biol.* **36**, 979–991 (2016).
50. J. S. Runge, J. R. Raab, T. Magnuson, Identification of two distinct classes of the human INO80 complex genome-wide. *G3 (Bethesda)* **8**, 1095–1102 (2018).
51. J. Bollard, V. Miguela, M. Ruiz de Galarreta, A. Venkatesh, C. B. Bian, M. P. Roberto, V. Tovar, D. Sia, P. Molina-Sánchez, C. B. Nguyen, S. Nakagawa, J. M. Llovet, Y. Hoshida, A. Lujambio, Palbociclib (PD-0332991), a selective CDK4/6 inhibitor, restricts tumour growth in preclinical models of hepatocellular carcinoma. *Gut* **66**, 1286–1296 (2017).
52. F. P. Reiter, G. Denk, A. Ziesch, A. Ofner, R. Wimmer, S. Hohenester, T. S. Schiergens, M. Spampatti, L. Ye, T. Itzel, S. Munker, A. Teufel, A. L. Gerbes, J. Mayerle, E. N. De Toni, Predictors of ribociclib-mediated antitumour effects in native and sorafenib-resistant human hepatocellular carcinoma cells. *Cell. Oncol. (Dordr.)* **42**, 705–715 (2019).
53. M. A. Horlbeck, L. A. Gilbert, J. E. Villalta, B. Adamson, R. A. Pak, Y. Chen, A. P. Fields, C. Y. Park, J. E. Corn, M. Kampmann, J. S. Weissman, Compact and highly active next-generation libraries for CRISPR-mediated gene repression and activation. *eLife* **5**, e19760 (2016).
54. J. G. Doench, N. Fusi, M. Sullender, M. Hegde, E. W. Vaimberg, K. F. Donovan, I. Smith, Z. Tothova, C. Wilen, R. Orchard, H. W. Virgin, J. Listgarten, D. E. Root, Optimized sgRNA design to maximize activity and minimize off-target effects of CRISPR-Cas9. *Nat. Biotechnol.* **34**, 184–191 (2016).
55. H. J. Uckelmann, S. M. Kim, E. M. Wong, C. Hatton, H. Giovinazzo, J. Y. Gadrey, A. V. Krivtsov, F. G. Rücker, K. Döhner, G. M. McGeehan, R. L. Levine, L. Bullinger, G. S. Vassiliou, S. A. Armstrong, Therapeutic targeting of preleukemia cells in a mouse model of *NPM1* mutant acute myeloid leukemia. *Science* **367**, 586–590 (2020).
56. A. Shevchenko, H. Tomas, J. Havlis, J. V. Olsen, M. Mann, In-gel digestion for mass spectrometric characterization of proteins and proteomes. *Nat. Protoc.* **1**, 2856–2860 (2006).

Acknowledgments

Funding: This work was supported by National Institutes of Health grant R37 CA233691 (C.-W.C.), National Institutes of Health grant R01 CA236626 (C.-W.C.), National Institutes of Health grant U54 CA243124 (C.-W.C.), Alex's Lemonade Stand Foundation Innovation grant (C.-W.C.), National Institutes of Health grant R01 CA271497 (J.C.), National Institutes of Health grant R01 CA243386 (J.C.), and Simms/Mann Family Foundation (J.C.). Research reported here included work performed in POSEIDON (Precision Oncology Software Environment Interoperable Data Ontologies Network), the Integrative Genomics Shared Resource, the Drug Discovery and Structural Biology Shared Resource, and the Integrated Mass Spectrometry Shared Resource supported by the National Cancer Institute of the National Institutes of Health grant P30 CA033572 (S.T.R.). **Author contributions:** Conceptualization: X.X., M.M., S.T.R., J.C., R.S., L.Y., and C.-W.C. Methodology: X.X., L.Y., A.K.N.C., S.P.P., J.H.W., P.P., Y.-C.Y., J.W., and C.-W.C. Investigation: X.X., S.P.P., M.L., Q.L., N.M., W.-H.C., J.H.W., R.E.M., P.P., Y.-C.Y., and J.W. Visualization: X.X., L.Y., A.K.N.C., and C.-W.C. Funding acquisition: J.C., S.T.R., and C.-W.C. Supervision: C.-W.C. Writing—original draft: X.X., L.Y., and C.-W.C. Writing—review and editing: X.X. and C.-W.C. **Competing interests:** J.C. is a scientific advisor for Race Oncology. The authors declare that they have no other competing interests. **Data and materials availability:** The RNA-seq and TST-captured genomic DNA-seq data generated in this study are available via Gene Expression Omnibus (GEO {https://www.ncbi.nlm.nih.gov/geo/}) at accession GSE199143. All data needed to evaluate the conclusions in the paper are present in the paper and/or the Supplementary Materials.

Submitted 6 May 2022
Accepted 10 November 2022
Published 23 December 2022
10.1126/sciadv.adc8911
