# Supplementary material for: ACTR5 controls CDKN2A and tumor progression in an INO80-independent manner
Source: Sci Adv. 2022 Dec 23;8(51):eadc8911. doi: 10.1126/sciadv.adc8911 (PMC9788768; doi:10.1126/sciadv.adc8911)
Supplement: Supplementary file 2 — Figs. S1 to S11 Tables S1 to S3 [file sciadv.adc8911_sm.pdf]

Science Advances
AAAS

Supplementary Materials for

ACTR5 controls CDKN2A and tumor progression in an INO80-independent manner

Xiaobao Xu *et al.*

Corresponding author: Chun-Wei Chen, cweichen@coh.org

Sci. Adv. **8**, eadc8911 (2022)
DOI: 10.1126/sciadv.adc8911

The PDF file includes:

Figs. S1 to S11
Tables S1 to S3
Legend for suppl excel data table

Other Supplementary Material for this manuscript includes the following:

Suppl excel data table

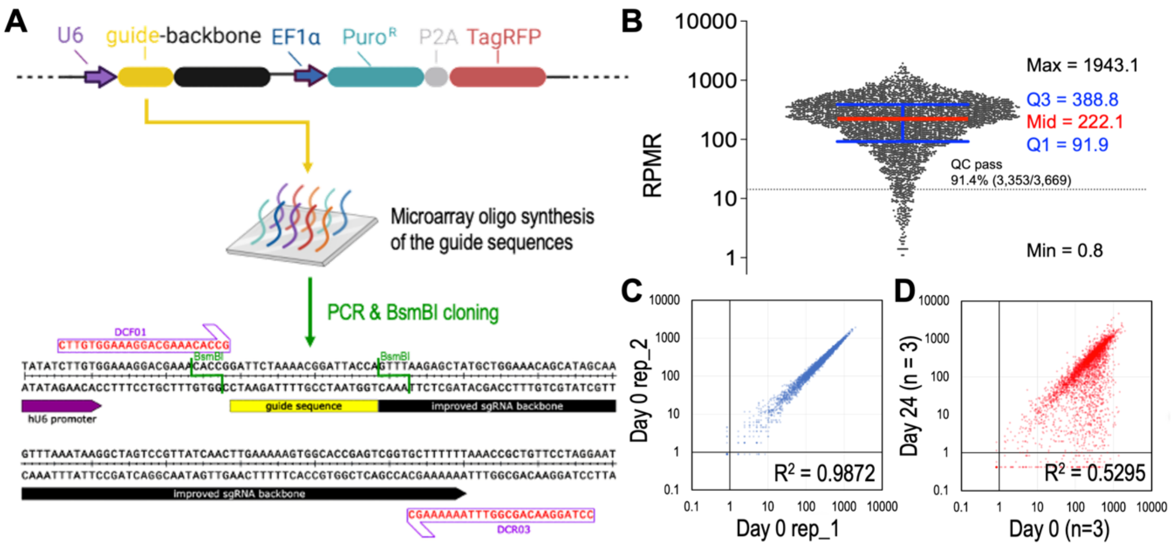

fig. S1. Epigenetic CRISPRi library.

(A) Map of the ipUSEPR vector expressing a sgRNA together with a puromycin-resistant gene (PuroR) and a TagRFP fluorescent protein. DCF01 and DCR03 primers for Illumina sequencing are listed. (B) Distribution of individual sgRNA frequencies (reads per million reads; RPMR) in the epigenetic CRISPRi library with 91.4% of sgRNAs passed the QC by exhibiting ≥ 5% of expected frequency. (C-D) Data correlation of individual sgRNAs of the epigenetic CRISPRi library between (C) two replications at day 0, and (D) the average of day 0 and day 24 (n = 3 each timepoint).

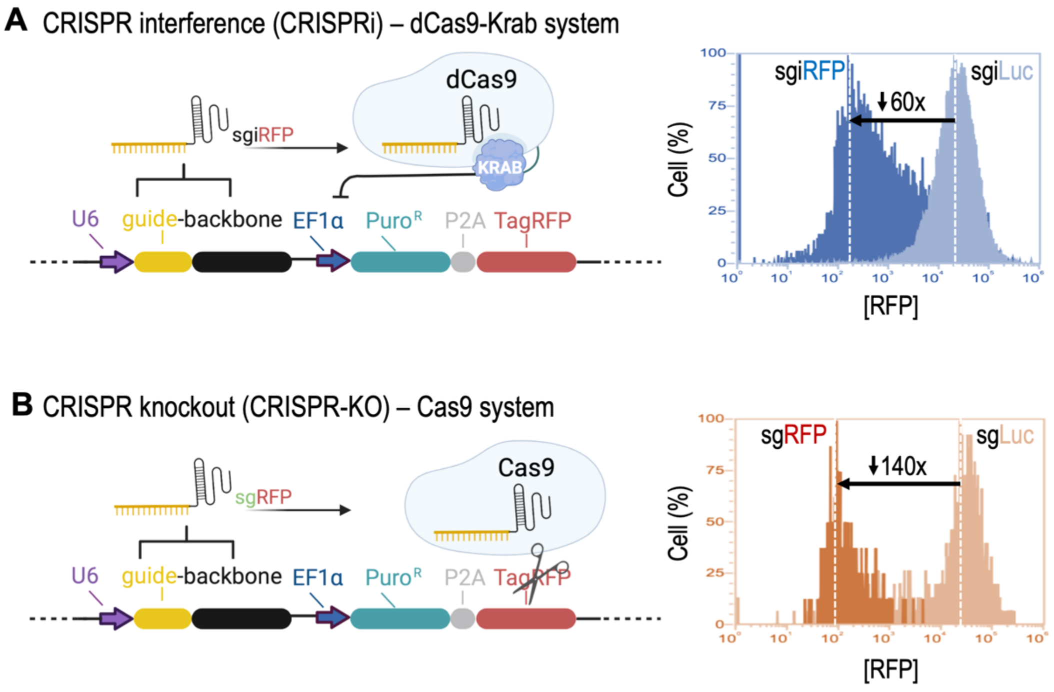

fig. S2. Gene silencing efficiency of the CRISPR systems.

The gene silencing efficiency of (A) CRISPR interference (CRISPRi) and (B) CRISPR knockout (CRISPR-KO) systems was measured by an RFP inactivation assay.

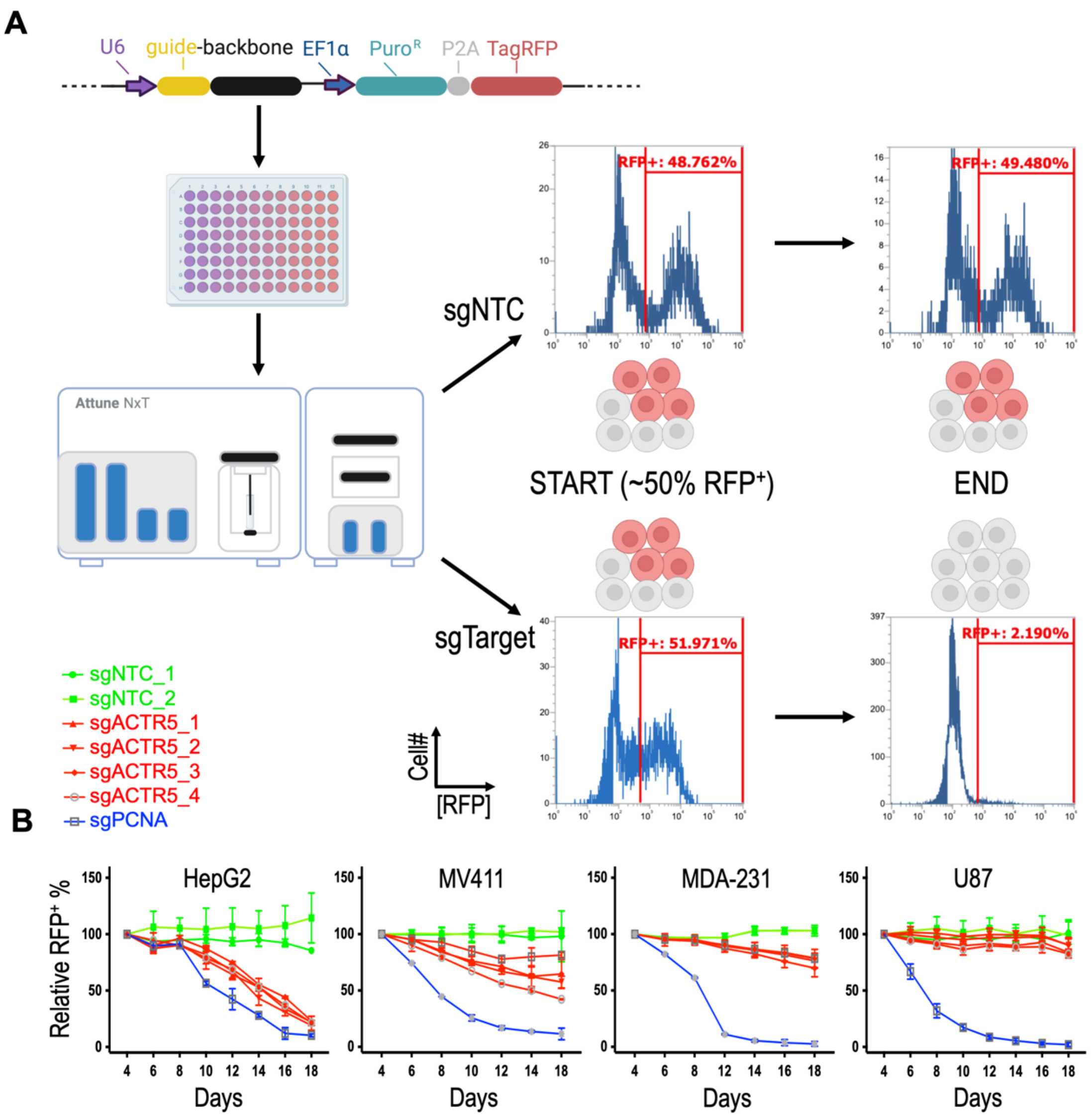

fig. S3. RFP flow cytometric growth competition assay.

(A) Schematic outline of the growth competition assay using the RFP expression labeling the sgRNA transduced cells in the pooled cultures. The RFP-positive % were measured in the DAPI-negative (live), singlet cell population of each sample using an Attune NxT flow cytometer with a 96-well autosampler. (B) Growth competition assay of Cas9-expressing HepG2 (HCC), MV411 (AML), MDA-231 (breast cancer), and U87 (GBM) cells transduced with the RFP-labeled sgNTC (green lines; n = 2 independent sequences), sgACTR5 (red lines; n = 4 independent sequences), and sgPCNA (blue lines; n = 1 sequence; PCNA is an essential gene in most cancer types).

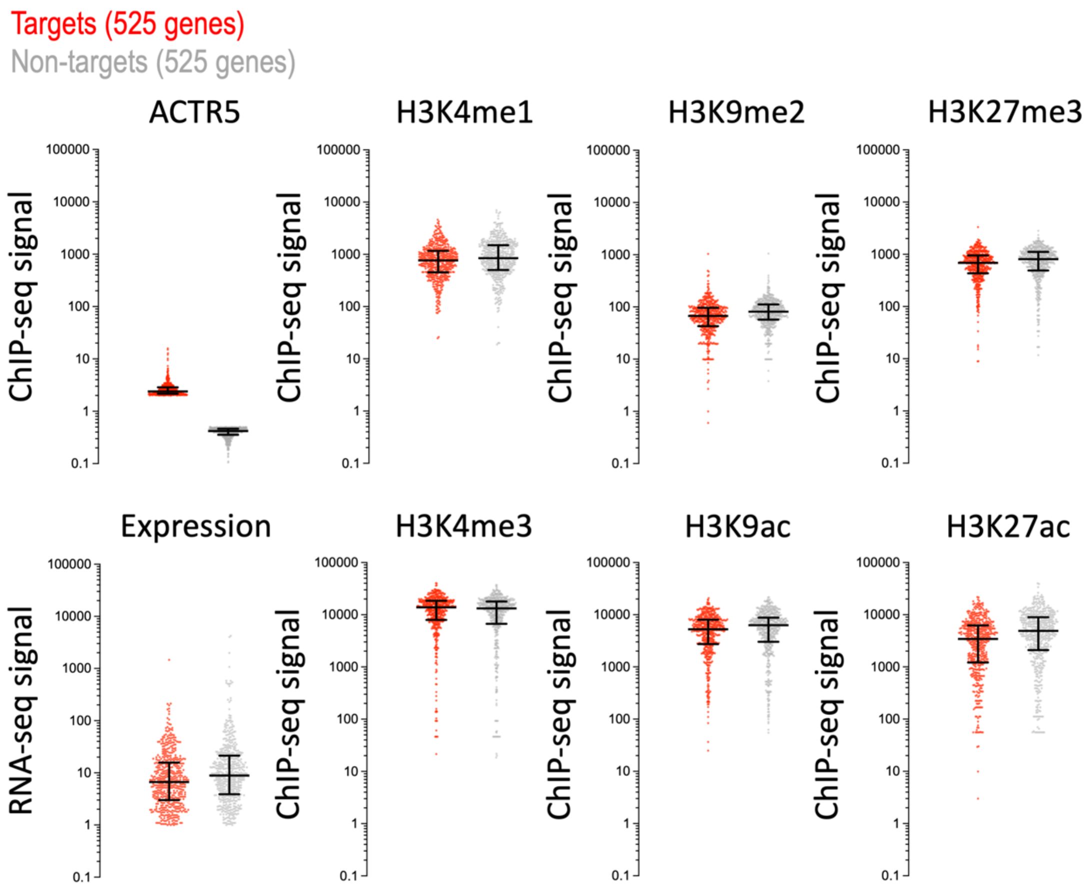

fig. S4. Comparison of the histone modification landscape at the ACTR5-bound and unbound genes.

The HepG2 histone modification ChIP-seq data were obtained from ENCODE Consortium (data source: B. Bernstein, BROAD Institute).

A GO analyses of the down-regulated 175 genes in sgiATCR5

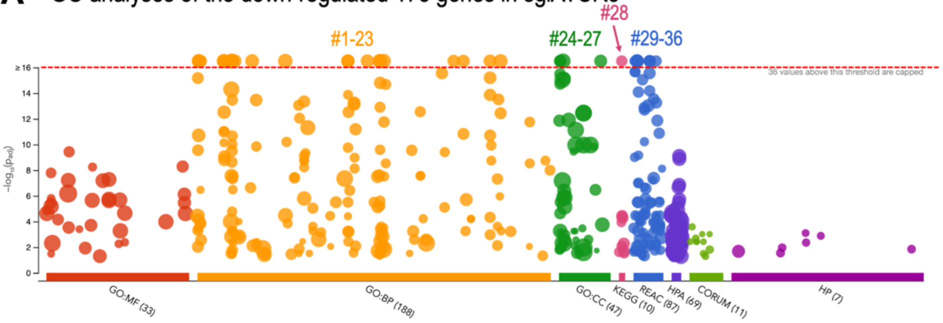

ID	Source	Term ID	Term Name	p_{adj} (query_1)
1	GO:BP	GO:0007049	cell cycle	1.896×10^{-53}
2	GO:BP	GO:0000278	mitotic cell cycle	8.946×10^{-50}
3	GO:BP	GO:1903047	mitotic cell cycle process	7.046×10^{-45}
4	GO:BP	GO:0022402	cell cycle process	1.142×10^{-44}
5	GO:BP	GO:0007059	chromosome segregation	5.119×10^{-35}
6	GO:BP	GO:0051301	cell division	9.842×10^{-33}
7	GO:BP	GO:0000280	nuclear division	3.412×10^{-31}
8	GO:BP	GO:0051276	chromosome organization	1.383×10^{-30}
9	GO:BP	GO:0048285	organelle fission	2.019×10^{-29}
10	GO:BP	GO:0051726	regulation of cell cycle	6.557×10^{-29}
11	GO:BP	GO:0098813	nuclear chromosome segregation	1.047×10^{-28}
12	GO:BP	GO:0010564	regulation of cell cycle process	9.795×10^{-27}
13	GO:BP	GO:0140014	mitotic nuclear division	1.717×10^{-26}
14	GO:BP	GO:0000819	sister chromatid segregation	3.150×10^{-25}
15	GO:BP	GO:0000070	mitotic sister chromatid segregation	2.587×10^{-24}
16	GO:BP	GO:0006259	DNA metabolic process	2.449×10^{-23}
17	GO:BP	GO:0006260	DNA replication	2.742×10^{-22}
18	GO:BP	GO:0006261	DNA-templated DNA replication	5.023×10^{-22}
19	GO:BP	GO:0044770	cell cycle phase transition	2.468×10^{-21}
20	GO:BP	GO:0007346	regulation of mitotic cell cycle	8.089×10^{-19}
21	GO:BP	GO:0044772	mitotic cell cycle phase transition	9.233×10^{-19}
22	GO:BP	GO:1901987	regulation of cell cycle phase transition	1.857×10^{-18}
23	GO:BP	GO:0006974	cellular response to DNA damage stimulus	1.043×10^{-17}
24	GO:CC	GO:0005694	chromosome	1.326×10^{-31}
25	GO:CC	GO:0098687	chromosomal region	5.637×10^{-29}
26	GO:CC	GO:0000793	condensed chromosome	1.648×10^{-21}
27	GO:CC	GO:0000775	chromosome, centromeric region	2.175×10^{-21}
28	KEGG	KEGG:04110	Cell cycle	9.252×10^{-22}
29	REAC	REAC:R-HSA-1640170	Cell Cycle	1.168×10^{-48}
30	REAC	REAC:R-HSA-69278	Cell Cycle, Mitotic	1.029×10^{-41}
31	REAC	REAC:R-HSA-69620	Cell Cycle Checkpoints	1.659×10^{-32}
32	REAC	REAC:R-HSA-2500257	Resolution of Sister Chromatid Cohesion	3.552×10^{-19}
33	REAC	REAC:R-HSA-68877	Mitotic Prometaphase	8.407×10^{-18}
34	REAC	REAC:R-HSA-176187	Activation of ATR in response to replication stress	1.350×10^{-17}
35	REAC	REAC:R-HSA-2555396	Mitotic Metaphase and Anaphase	2.228×10^{-17}
36	REAC	REAC:R-HSA-453279	Mitotic G1 phase and G1/S transition	2.503×10^{-17}

B GO analyses of the up-regulated 109 genes in sgiATCR5

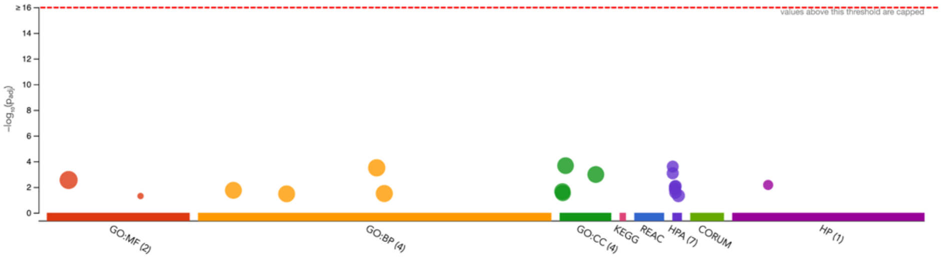

fig. S5. Gene ontology analyses of the ACTR5-regulated genes in HepG2 cells.

Gene ontology analyses (g:Profiler) of the down-regulated (A, 175 genes) and up-regulated genes (B, 109 genes) in sgiACTR5 transduced HepG2 cells over the following databases: (1) GO molecular function (GO:MF), (2) GO biological process (GO:BP), (3) GO cellular component (GO:CC), (4) Kyoto Encyclopedia of Genes and Genomes (KEGG), (5) Reactome (REAC), (6) Human Protein Atlas (HPA), (7) CORUM, and (8) Human Phenotype (HP).

A GO analyses of the down-regulated 175 genes in sgiATCR5

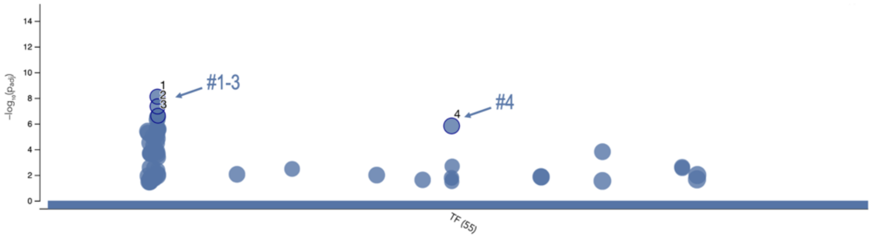

ID	Source	Term ID	Term Name	p_{adj} (query_1)
1	TF	TF:M00919_1	Factor: E2F; motif: NCSCGCSAAAN; match class: 1	7.889×10^{-9}
2	TF	TF:M00939_1	Factor: E2F; motif: TTTSGCGSG; match class: 1	4.517×10^{-8}
3	TF	TF:M00920_1	Factor: E2F; motif: NKCGCGCSAAAN; match class: 1	2.392×10^{-7}
4	TF	TF:M12642	Factor: NFYA; motif: NNRGCCAATSRGMRSSNNSN	1.492×10^{-6}

B GO analyses of the up-regulated 109 genes in sgiATCR5

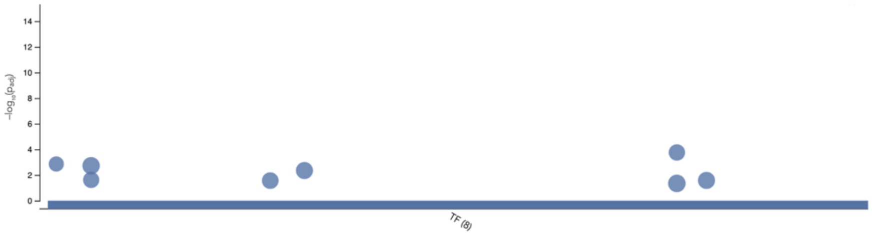

fig. S6. TRANSFAC analyses of the ACTR5-regulated genes in HepG2 cells.

Gene ontology analyses (g:Profiler) of the down-regulated (A, 175 genes) and up-regulated genes (B, 109 genes) in sgiACTR5 transduced HepG2 cells over the TRANSFAC database (TF; a database on transcription factors and their DNA-binding sites).

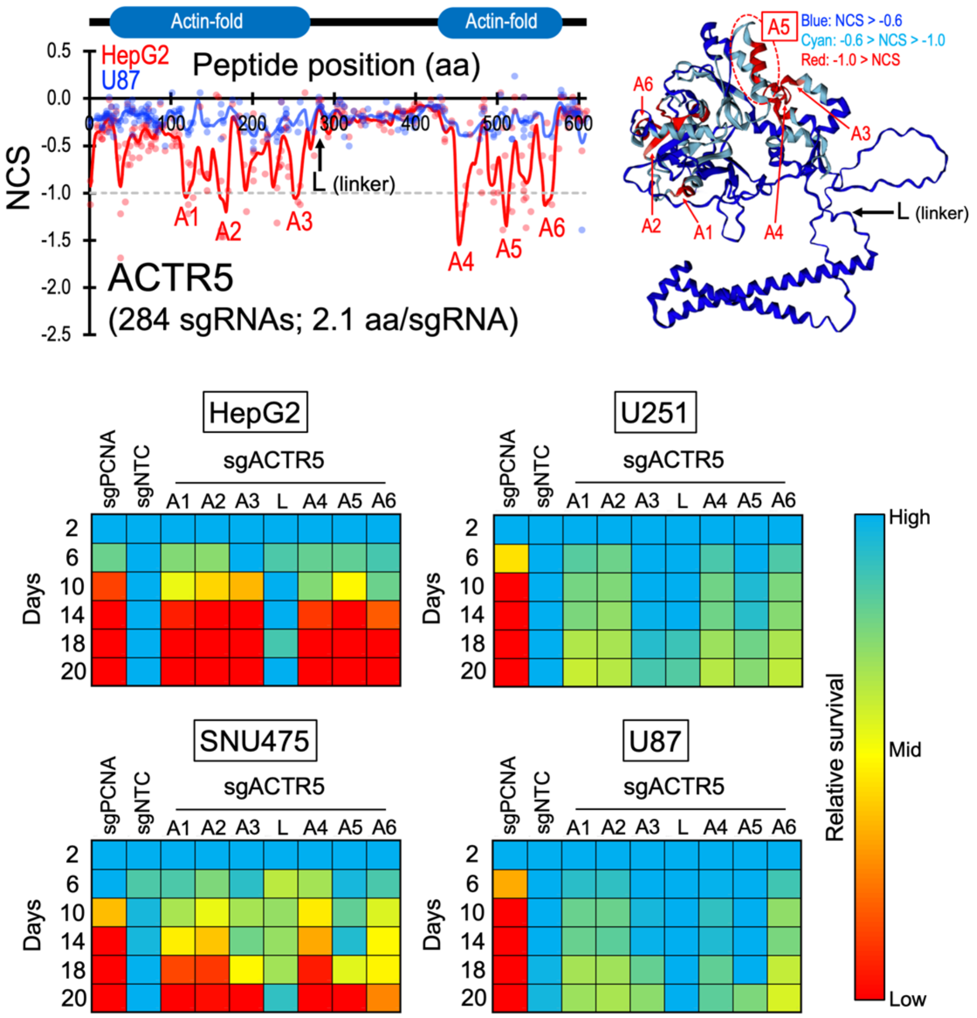

fig. S7. Validation of the CRISPR hypersensitive elements identified by CRISPR tiling scan.

(A) Schematic outline of the CRISPR hypersensitive elements (A1 – A6) and a linker region (L) in ACTR5.

(B) Growth competition assay of Cas9-expressing HepG2, SNU475, U251, and U87 cells transduced with sgRNAs targeting individual regions of ACTR5 (sgACTR5), a non-essential sequence (sgNTC), and an essential gene (sgPCNA).

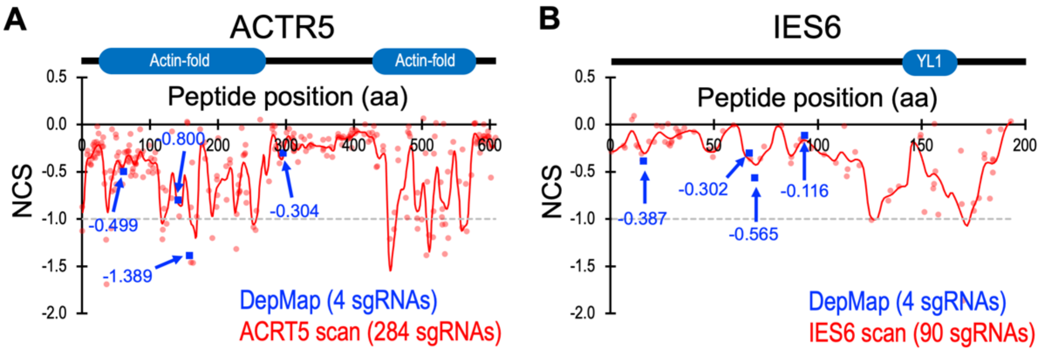

fig. S8. Comparison of sgRNAs used in DepMap and CRISPR gene tiling scan.

Two-dimensional annotation of (A) ACTR5 and (B) IES6 sgRNAs used in the DepMap genome-wide CRISPR screen project (blue; 4 sgRNAs) vs. the CRISPR gene tiling scans used in this study (red; 284 sgRNAs for ACTR5 and 90 sgRNAs for IES6). The red lines indicate the smoothened model of the CRISPR scan score derived from individual sgRNAs (dots) screened in HepG2 cells. The median CRISPR scan scores of the positive control (dotted line; defined as -1.0) and negative control (defined as 0.0) sgRNAs are designated.

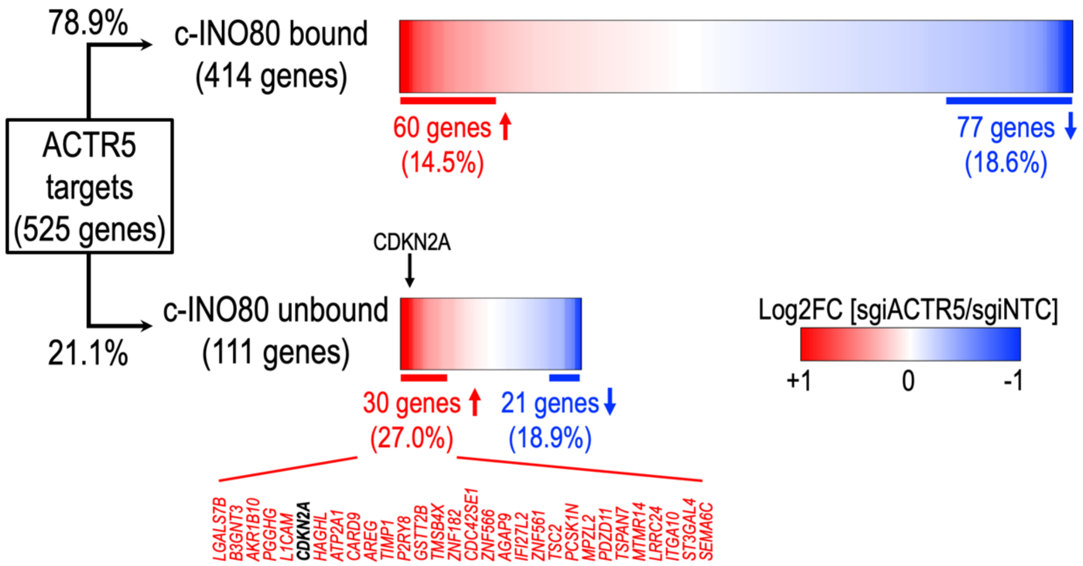

fig. S9. Differential regulation of canonical INO80 complex-bound and unbound ACTR5 target genes. Cross analysis of ACTR5-TST ChIP-seq targets (525 genes) with the canonical INO80 complex (c-INO80; defined by the previously reported INO80 and YY1 ChIP-seq in HepG2 cells)(*50*) bound loci revealed 21.1% of ACTR5 targets (111 genes) were not recognized by c-INO80. A higher proportion of these c-INO80-independent ACTR5 target genes (27.0% vs. 14.5% in the c-INO80 bound ACTR5 targets) showed increased expression upon CRISPR knockdown of ACTR5 in HepG2 cells.

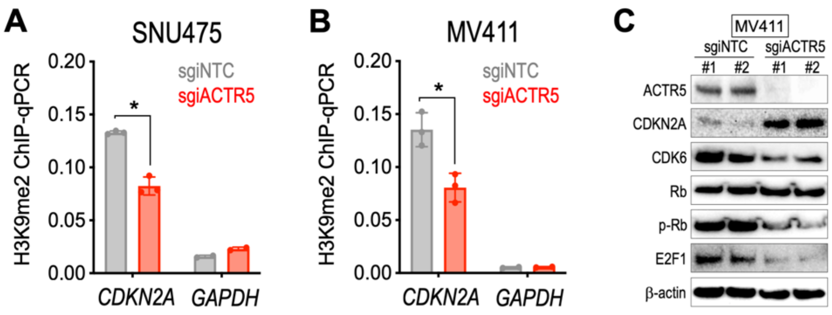

fig. S10. Effect of ACTR5-depletion on SNU475 and MV411 cells.

Level of H3K9me2 at the CDKN2A and GAPDH loci in (A) SNU475 and (B) MV411 cells transduced with sgiNTC and sgiACTR5. (C) Western blot of ACTR5, CDKN2A, CDK6, Rb, p-Rb, E2F1, and β-actin in MV411 cells transduced with sgiNTC and sgiACTR5. Data are represented as mean ± SEM. *P < 0.01 by two-sided Student's t-test.

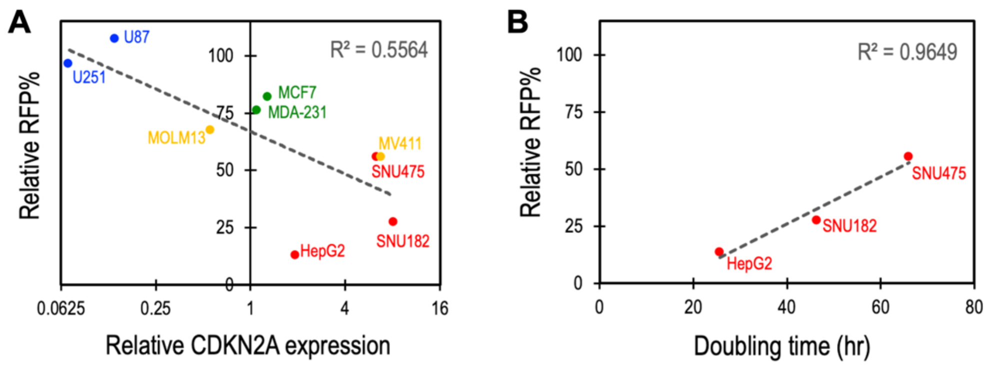

fig. S11. Correlation of the ACTR5-dependency with CDKN2A expression and proliferation index. Relationships between (A) relative CDKN2A expression (RNA-seq data obtained from the Cancer Cell Line Encyclopedia [CCLE]) and (B) cellular doubling time (obtained from Cellosaurus and ATCC) with the cellular response to ACTR5-depletion (relative RFP% for each cell model summarized from Fig. 1E).

table S1. CRISPRi sgiRNA sequences.

sgiRNA_ID	Sequence
sgiNTC_1	GATTCTAAAACGGATTACCA
sgiNTC_2	GGATGATAACTGGTCCGCAG
sgiATCR5_1	GACTGGGTCCGGTGCGGCAC
sgiATCR5_2	GGGCACGGGCGTCGCGGAAC
sgiATCR5_3	GGACGCGCGCTCCAAGATGG
sgiATCR5_4	GTCCAGCACTGGGTCCGGTG
sgiIES6_1	GAAGACTCGGAGTGCGATGG
sgiIES6_2	GAAGTTCCAAGGCCCGCGCT
sgiIES6_3	GCCCGCGCTGGGAAAAAGGT
sgiIES6_4	GATCAAGACGGAAGTAACAG

table S2. CRISPR-KO sgRNA sequences.

sgRNA_ID	Sequence
sgNTC_1	ATGTCGCCATAAATAAGAAG
sgNTC_2	TGTGCCAGAGTCCTTCGATA
sgATCR5_1	GGACTACAGCTTCCAGCACC
sgATCR5_2	TATTGTGGTAGAAGCTGAAG
sgATCR5_3	TGTTCTTCTCCTATGAGAGA
sgATCR5_4	ATTTTCCAGCCATCTCTCAT
sgPCNA	GGACTCGTCCCACGTCTCTT

table S3. ChIP-qPCR primer sequences.

Primer_ID	Sequence
CDKN2A TSS_F	TCTGTTAAAAAGAAATCCGCC
CDKN2A TSS_R	ACAACTAGGAAAGAATAGTTTTGC
HBB TSS_F	ATGCAGAGATATTGCTATTGCCTT
HBB TSS_R	TGTATCATGCCTCTTTGCACCA
GAPDH TSS_F	CTCGAGGAGAAGTTCCCCAAC
GAPDH TSS_R	GTCAAGGACGGGGACCCTTA

Suppl Excel data table
adc8911_Suppl. Excel_seq1_v2.xlsx
